# Hydroperoxidation of Docosahexaenoic Acid by Human ALOX12 and pigALOX15-mini-LOX

**DOI:** 10.3390/ijms24076064

**Published:** 2023-03-23

**Authors:** Miquel Canyelles-Niño, Àngels González-Lafont, José M. Lluch

**Affiliations:** 1 Departament de Química, Universitat Autònoma de Barcelona, Bellaterra, 08193 Barcelona, Spain; 2 Arquebio SL, Carrer de Álava 51, 08005 Barcelona, Spain; 3 Institut de Biotecnologia i Biomedicina (IBB), Universitat Autònoma de Barcelona, Bellaterra, 08193 Barcelona, Spain

**Keywords:** hydroperoxidation mechanism, human platelet ALOX12, enzyme catalysis, molecular dynamics simulations, QM/MM calculations

## Abstract

*Human* lipoxygenase 12 (hALOX12) catalyzes the conversion of docosahexaenoic acid (DHA) into mainly 14S-hydroperoxy-4Z,7Z,10Z,12E,16Z,19Z-docosahexaenoic acid (14S-H(p)DHA). This hydroperoxidation reaction is followed by an epoxidation and hydrolysis process that finally leads to maresin 1 (MaR1), a potent bioactive specialized pro-resolving mediator (SPM) in chronic inflammation resolution. By combining docking, molecular dynamics simulations, and quantum mechanics/molecular mechanics calculations, we have computed the potential energy profile of DHA hydroperoxidation in the active site of hALOX12. Our results describe the structural evolution of the molecular system at each step of this catalytic reaction pathway. Noteworthy, the required stereospecificity of the reaction leading to MaR1 is explained by the configurations adopted by DHA bound to hALOX12, along with the stereochemistry of the pentadienyl radical formed after the first step of the mechanism. In *pig* lipoxygenase 15 (pigALOX15-mini-LOX), our calculations suggest that 14S-H(p)DHA can be formed, but with a stereochemistry that is inadequate for MaR1 biosynthesis.

## 1. Introduction

Mammalian lipoxygenases (LOXs) form a heterogeneous family of non-heme iron-containing enzymes that catalyze the dioxygenation of polyunsaturated fatty acids (PUFAs) with cis-methylene-interrupted double bonds. The peroxidation process is highly regio- and stereospecific, leading to the corresponding conjugated hydroperoxides [1]. Then, along the biological pathway, those hydroperoxy fatty acids formed by the different LOXs isoforms generate a wide variety of bioactive lipid mediators that are involved in a plethora of pro- and anti-inflammatory processes [1,2,3].

The first lipoxygenase discovered in animal tissues by Nobel Laureate Samuelsson and his colleague Hamberg was the *human* 12-lipoxygenase of blood platelets [4]. This enzyme was named 12S-LOX because it oxidizes arachidonic acid (AA) to generate almost exclusively one regio- and stereospecific isomer, the 12S-hydro(per)oxyeicosa-5,8,10,14-tetraenoic acid (12S-H(p)ETE) [5]. However, this nomenclature was confusing because the regio- and stereospecificity of LOX isoforms depend on the substrate and are species-specific [6,7]. Moreover, this nomenclature is not related to the biological function of LOX isoforms. For this reason, terminology based on the gene encoding each LOX isoform is more recommended. In humans, the platelet-type 12-LOX (12S-LOX) is encoded by the *ALOX12* gene and is named *human* ALOX12 (hALOX12) [7]. The hALOX12 peroxidation reaction is initiated by H-abstraction of the H_10_ atom of AA by the Fe(III)-OH^−^ cofactor. Next, an oxygen molecule inserts on C_12_ following an antarafacial approach and gives a hydroperoxide with S stereochemistry. Finally, the reduced product, 12S-HETE, is obtained by the action of glutathione peroxidase.

*Human* ALOX12 is the unique LOX isoform expressed in *human* platelets [4]. Nowadays this LOX has been found in platelets of several other mammals such as *bovine*, *mouse*, *rat*, *rabbit*, *sheep*, and *dog* [8,9,10,11] but is not expressed in *porcine* platelets [12]. Through hALOX12 oxidized metabolites, this enzyme regulates platelet activity in hemostasis and thrombosis, and controls platelet role in inflammatory processes. According to several studies with *human* patients, hALOX12 and its derived oxylipin 12S-HETE seem to present prothrombotic effects [13]. However, the prothrombotic role of 12S-HETE in vivo is not fully understood yet. There are contradictory experimental results with animal models in which 12S-HETE could either promote or inhibit or even not participate in platelet aggregation [14,15]. In this respect, the first selective hALOX12 inhibitor, ML355, has been shown to impair thrombus formation and vessel occlusion in vivo with minimal effects on hemostasis [16,17]. It has also been recognized that platelets are involved in the pathogenesis of several diseases, such as asthma, cancer, neurodegenerative disorders, gastrointestinal and hepatic inflammation, insulin resistance, and atherosclerosis, in which chronic inflammation plays an underlying role [7,18]. So, 12-HETE has been described as a pro-inflammatory molecule. However, it is not always clear whether the 12-HETE present in the inflamed tissue originated from platelets or from other cells because hALOX12 is also expressed in leucocytes, *human* islets, and epidermal keratinocytes [19]. On the other hand, hALOX12, in conjunction with other LOX isoforms, is involved in the transcellular biosynthesis of AA-derived anti-inflammatory agents such as lipoxins [20].

*Human* ALOX12 also catalyzes the oxygenation of the ω-3 fatty acid 4,7,10,13,16,19-docosahexaenoic acid (DHA). DHA can be converted by hALOX12 into two different products, depending on the experimental conditions. In this respect, there are different results in the literature concerning the regioselectivity of the DHA oxygenation reaction with hALOX12. According to Kühn and co-workers [21], the ω-9 hydroperoxide product 14-hydroperoxydocosahexaenoic acid (14-H(p)DHA) is dominant over DHA. Those experimental results did not show significant amounts of any other product with this enzyme. Thus, hALOX12 catalysis with DHA was classified as singular ω-9 oxygenase activity. Two years later, Holman and co-workers [22] published new kinetic results that confirmed 14-H(p)DHA as the main product of DHA peroxidation by hALOX12. However, the obtained percentage of 11-HDHA:14-HDHA (29:71) products (the reduced forms of 11-hydroperoxydocosahexaenoic acid (11-H(p)DHA) and 14-H(p)DHA, respectively) was higher than previously reported. According to the authors, those differences appear because they expressed hALOX12 in *insect* cells, whereas Kühn and co-workers used E. coli as the expression system [21]. In a following paper, Holman and co-workers report a smaller in vitro proportion of 11S-HDHA (19%) to 14S-HDHA (81%), and an even smaller ex vivo percentage (6:94) [23]. The DHA peroxidation mechanism is proposed to initiate with an H-abstraction reaction (described as a proton-coupled electron transfer, PCET [24,25]) of H_12_ or H_9,_ in this case, by the Fe(III)-OH^₋^ cofactor. Next, an oxygen molecule inserts on C_14_ or C_11_ of DHA, following an antarafacial approach, and gives the corresponding hydroperoxides with S stereochemistry, which are immediately reduced to 14-HDHA and 11-HDHA, respectively. Moreover, steady-state kinetic measurements of this catalytic mechanism of hALOX12 have demonstrated that the rates of substrate capture and product release are similar between DHA and AA [23,26].

The biological relevance of DHA-derived oxylipins, 11-HDHA and 14-HDHA, is currently a focus of interest. The cardiovascular benefits of a diet rich in ω-3 fatty acids such as DHA have been known for decades, but the mechanisms involved in their anti-aggregatory effects on platelets are still under study. Recent in vitro and ex vivo experiments [27] with *mice* and *human*-washed platelets have confirmed that DHA, as well as 11-HDHA and 14-HDHA, inhibit collagen-induced platelet aggregation. Interestingly, the in vivo thrombosis data in *mice* showed that only acute concentrations of 11-HDHA or 14-HDHA attenuated overall thrombus formation. This fact suggests that under physiological conditions (at similar basal ALOX12-derived oxylipin levels than those used in the assays mentioned), 11-HDHA and 14-HDHA may play a central role in the regulation of thrombus formation by activating Protein Kinase A (PKA). Nevertheless, the authors of the experimental study do not discard additional complex mechanisms driven by specialized pro-resolving mediators (SPMs), also derived from DHA, that could contribute to antithrombotic effects. Maresin 1 (7R,14S-dihydroxy-4Z,8E,10E,12Z,16Z,19Z- docosahexaenoic acid, MaR1) is an SPM derived from 14S-H(p)DHA that is subsequently converted to 13S,14S-epoxy-4Z,7Z,9E,11E,16Z,19Z-docosahexaenoic acid through an epoxidation mechanism. Then, this epoxide intermediate is proposed to be enzymatically hydrolyzed to form the stereochemistry of bioactive MaR1 [23,28]. MaR1 is a potent SPM that actively participates in the inflammation-resolution phase of many different diseases, and it has also been described as a novel antiplatelet agent crucial in the resolution of inflammation in cardiovascular injuries [29,30].

At the molecular level, the structural basis of hALOX12's product selectivity has been much less studied than in the case of *rabbit* and *human* ALOX15. Remarkably, some mutagenesis experiments back in 2010 already showed that the triad concept (which established that the three residues, Phe353, Ile418, and Phe593, at the bottom of *rabbit* ALOX15's cavity were critical for AA positioning) only partially applies to hALOX12 [31,32]. Instead, this hypothesis has been supported several times for ALOX15 orthologs. By mutagenesis of the residues of the triad, an AA 15-lipoxigenating ALOX15 ortholog has been converted into an AA 12-lipoxygenating enzyme or vice versa. However, a U-shaped binding pocket [33,34,35] was introduced to complete the description of the substrate binding cavity of LOX isoforms versus the triad concept [6]. Moreover, the regioselectivity of LOX isoforms has also been shown to depend on the orientation of the substrate bound to the enzyme. That is, which end of the substrate (carboxylate-head or hydrophobic-tail) is innermost in the cavity [6,35]. Recently, Holman and co-workers [22] have thoroughly studied, by site-directed mutagenesis and, using docking calculations, the main interactions of AA with hALOX12 active-site residues, establishing the main differences between hALOX12 and hALOX15 regioselectivities. In that work, the double mutant A417I/V418M of hALOX12 increased 15-HpETE production by only 24 ± 2%, whereas the single mutation of hALOX15 (I418A) augmented the yield of 12-HETE to 94% [31]. Also, the docking simulations by Holman and co-workers supported the U-shaped binding mode [35] for AA in hALOX12 with a tail-first orientation [22]. However, the calculations in that paper were performed using a model of the catalytic domain of hALOX12 built from the crystallographic coordinates of an N-terminal truncation variant of pigALOX15 [36], so-called pigALOX15-mini-LOX. As the same authors explain, the X-ray structure for hALOX12‘s catalytic domain, available at the Protein Data Bank (PDB code 3D3L), is not adequate for modeling the AA/hALOX12 complex.

The *porcine* LOX isoform is encoded by the *ALOX15* gene, and for that reason, it is called pigALOX15. It presents a high degree (86%) of sequence conservation with rabALOX15, but much less similarity with hALOX12 (66%). This *porcine* LOX was purified from the cytosolic fraction of leukocytes [37], and it was later also mainly found in the anterior pituitary gland of *pigs* [38]. As with the other ALOX15 orthologs, pigALOX15 presents dual specificity when reacting with AA. The main pigALOX15 peroxidation products are 12S- and 15S-HpETE in a ratio of about 10:1 [39,40]. However, as mentioned above, this regioselectivity has been inverted by site-directed mutagenesis based on the triad concept. In a study by some of the authors [41], the complete wild-type pigALOX15 and its pigALOX15-mini-LOX variant were expressed, and both showed the same regiospecificity, mainly producing 12-H(p)ETE. However, catalytic activity was strongly reduced for the isolated catalytic domain. The specificity switch of pigALOX15-mini-LOX induced by experimental mutagenesis of Val418 and Val419 sequence determinants was demonstrated. Moreover, docking, MD simulations, and quantum mechanics/molecular mechanics calculations using a solvated model of wild-type and the Val418Ile + Val419Met double mutant of pigALOX15-mini-LOX were performed. In agreement with experiments, the lower energy barriers in the wild-type LOX were found for the H_10_-abstraction process leading to 12-lipoxygenation whereas the double mutant was dominantly 15-lipoxygenating with lower barriers for the H_13_-abstraction.

In this study, we have built for the first time an in silico model of the DHA/hALOX12 complex using the AlphaFold [42,43] server to retrieve the initial coordinates of the *human* LOX protein. Docking and MD simulations have been performed to simulate the binding mode of DHA at the active site of hALOX12. Next, we have calculated within a QM/MM scheme the potential energy profile of the hydroperoxidation mechanistic steps that convert DHA to the fully characterized 14S-hydroperoxy-4Z,7Z,10Z,12E,16Z,19Z-docosahexaenoic acid (14S-H(p)DHA). Special attention has been devoted to the stereochemistry of the reaction because the biosynthesis of maresin 1, which is the potent bioactive SPM derived from 14S-H(p)DHA, is highly stereospecific. For the sake of comparison, the viability of DHA peroxidation by pigALOX15 has also been analyzed using the crystallographic coordinates of an N-terminal truncation variant of pigALOX15 (pigALOX15-mini-LOX) that has been previously used as a model of the hALOX12 structure.

## 2. Results and Discussion

Docking calculations were carried out for the DHA/hALOX12 and DHA/pigALOX15-mini-LOX complexes. The binding mode of the substrate with the best score in the active site of each LOX was selected to initiate the corresponding MD simulations.

### 2.1. MD Simulations of the DHA/hALOX12 Complex

**Figure 1:**
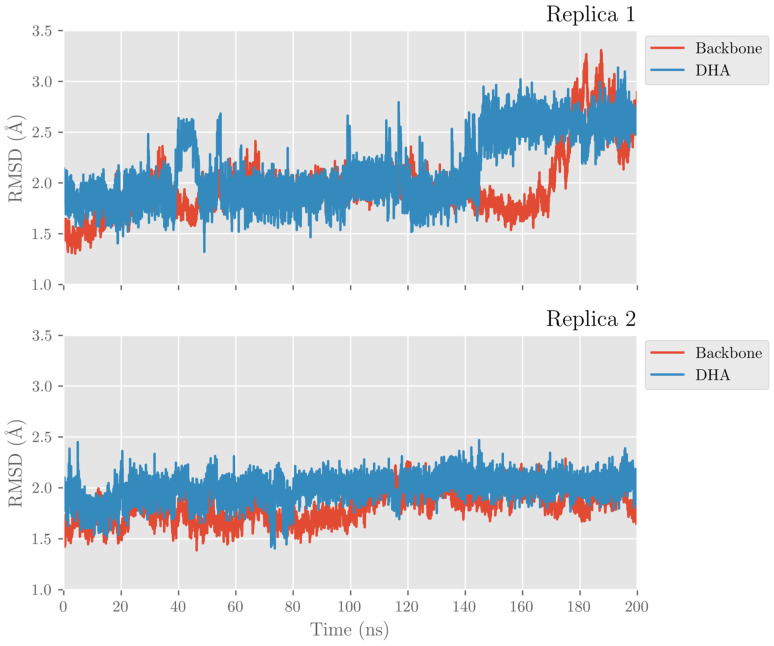
Protein and substrate backbone RMSDs versus time for the MD replica 1 and replica 2 in hALOX12.

**Figure 2:**
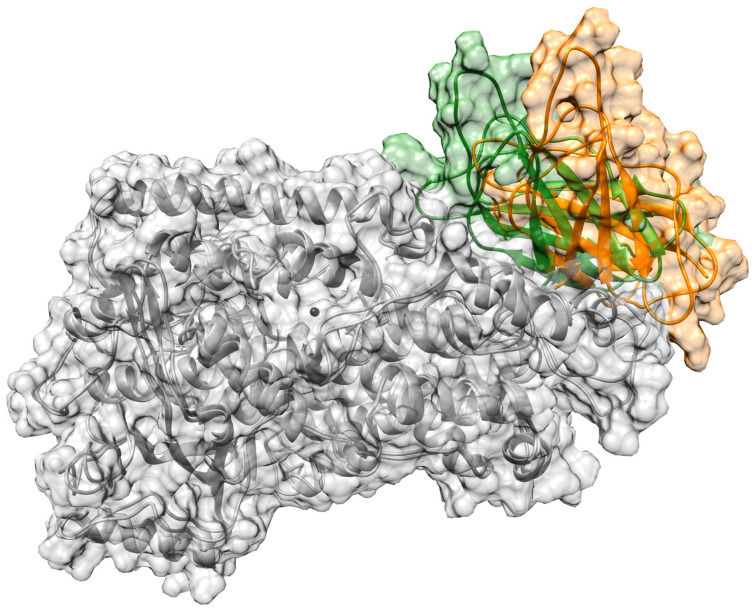
PLAT domain of hALOX12 in the closed (in green) and in the open (in orange) conformation. The C-terminal domain is shown in grey.

In this section, the achieved outcomes from the two MD trajectories of the solvated DHA/hALOX12 complex are presented. In Figure 1, we have plotted the protein backbone RMSDs versus time. The protein RMSD along the first replica remains quite stable during 170 ns. Then, a conformational change occurs that increases the RMSD. This conformational change corresponds to an opening of the PLAT (Polycystin-1,Lipoxygenase, Alpha-Toxin) domain (N-terminal domain from residues 1–110) away from the catalytic domain (C-terminal domain) (Figure 2). The average distance between the geometrical centers of the two domains changes from 42.3 Å to 47.2 Å when the domains separate (see Figure S1). The angle between the longest axes of the two ellipsoids that enclose the PLAT and the catalytic domain, respectively, is 38.2° in the closed conformation and 58.8° in the open structure. Experimentally, the flexibility of the PLAT domain of hALOX12, described as a pendulum-like movement, was proposed for fitting SAXS measurements [44,45]. The C-skeleton of DHA also experiences a sudden conformational change just before the N-terminal displacement (see Figure 1, replica 1). In the second MD replica, the PLAT domain remains more tightly bound to the catalytic domain in a close conformation (see Figure 1, replica 2). Moreover, in this second MD replica, the DHA substrate shows the same binding mode all along the trajectory.

**Figure 3:**
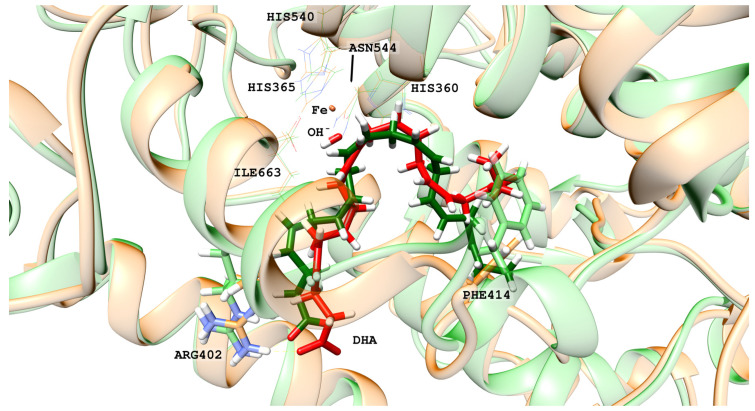
DHA binding mode in the closed (in green) and the open (in red) conformations of hALOX12. The interaction between Arg402 and the carboxylate group of DHA is shown as well as the rotation of Phe414.

In the hALOX12 active site, DHA adopts a U-shaped binding mode [35], like that described for AA by Holman and co-workers using docking calculations [22], while the two-domain LOX remains closed. There is a modification of the DHA pose bound to hALOX12 when the PLAT domain separates. An overlay of both DHA binding modes is depicted in Figure 3.

**Figure 4:**
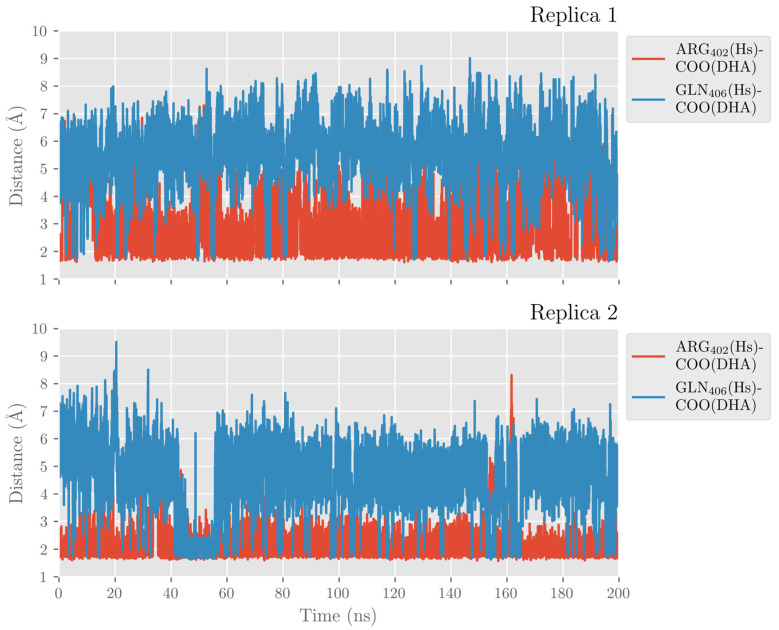
Distances of a carboxylate’s oxygen atom of DHA to the closest hydrogen atom in Arg402 and Gln406 versus time for the MD replica 1 and replica 2 in hALOX12.

In Figure 4, the interatomic distances of the main interactions between the carboxylate head of DHA and hALOX12 residues have been plotted along the two MD trajectories. As DHA has two more carbons than AA, its carboxylate group is hydrogen-bonded to Arg402 instead of being bound to His596 like AA (see Figure 3). Arg402 is a more solvent-exposed residue than His596 that can only be reached by DHA being a longer substrate. The average hydrogen-bond distance between Arg402 and the carboxylate of DHA (2.41 Å and 2.12 Å in replicas 1 and 2, respectively) is similar in both MD replicas, but in replica 2, the interaction remains more stable all along the trajectory.

**Figure 5:**
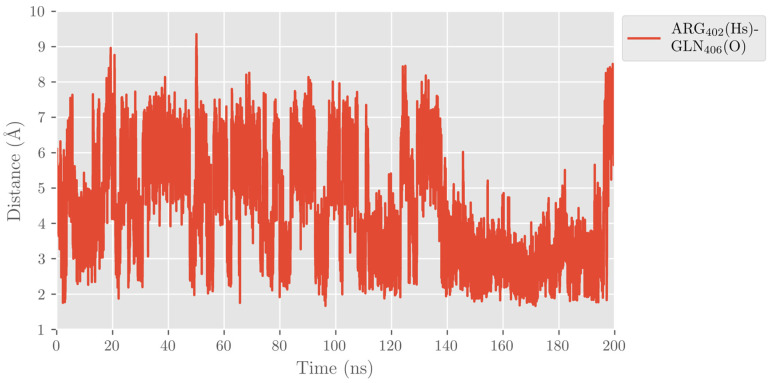
Distance between the oxygen atom in Gln406 and the closest hydrogen atom in Arg402 versus time for the MD replica 1 in hALOX12.

The carboxylate group of DHA also interacts with Gln406, but this interaction is less favorable and more fluctuating than with Arg402. In turn, Arg402 and Gln406 interact by hydrogen bonds along the MD trajectory, as can be seen in Figure 5 for replica 1. However, Gln406 flips repeatedly away from the cavity, losing contact with DHA’s carboxylate and Arg402. In contrast, Arg402 remains more fixed along the trajectory due to its electrostatic interactions with Glu175 of the α2-helix. Several water molecules also contribute to stabilizing the substrate’s carboxylate head.

Holman and co-workers mutated the Arg402 residue by a hydrophobic Leu and did not observe any significant change in the reactivity of hALOX12 with DHA [22]. However, this result does not necessarily mean that Arg402 does not interact with the carboxylate group of DHA in WT hALOX12, as was suggested by the authors of the mutagenesis experiment. In the Arg402Leu mutant, the DHA carboxylate group might reinforce its interaction with Gln406 and the closer water molecules. Thus, the change of hydrogen-bonded partner (mainly Arg402 in WT hALOX12 by Gln406 in the Arg402Leu mutant) would not imply any relocation of DHA.

**Figure 6:**
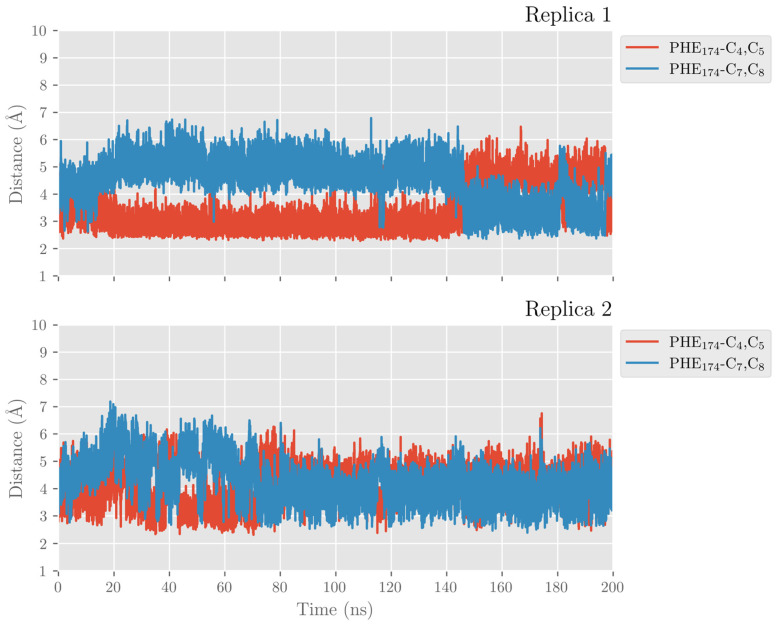
Distances between Phe174 side chain and the closest atom of the Δ^4^ and Δ^7^ double bonds versus time for the MD replica 1 and replica 2 in hALOX12.

**Figure 7:**
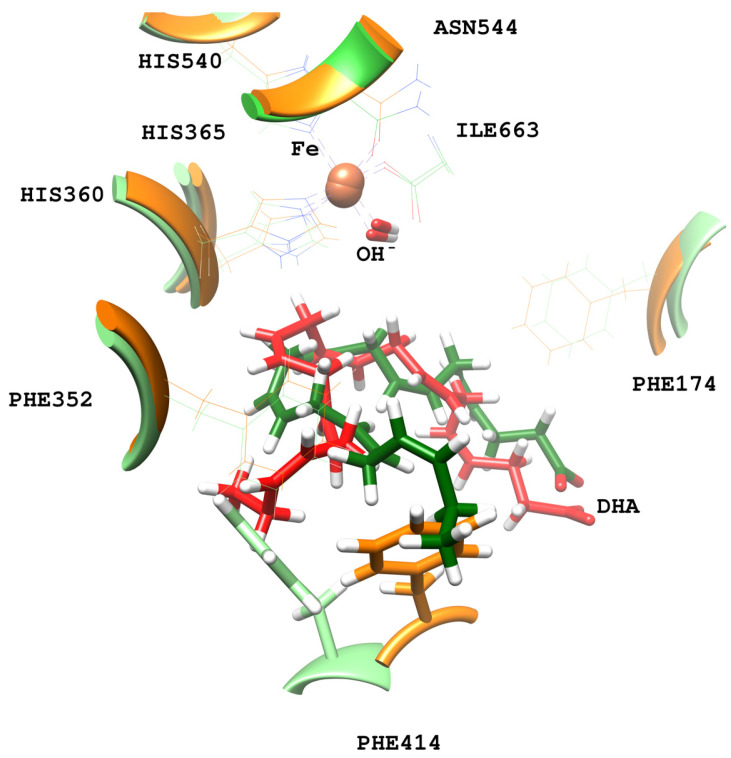
Main stacking interactions between hALOX12 residues and DHA. The protein residues and DHA are shown in green for the closed conformation, and in orange and red, respectively, for the open conformation of the MD replica 1.

DHA also establishes π–π interactions between its six double bonds and aromatic residues of the hALOX12 active site that stabilize the substrate binding mode. In Figure 6, we plotted the distances between Phe174 and the closer carbon atom of Δ^4^ and Δ^7^ double bonds along the MD trajectory of replica 1. This π–π stacking stabilizes the Δ^4^ double bond during the first 150 ns of the trajectory until the DHA substrate moves to the cavity entrance. Then, Phe174 switches its stacking interactions from the Δ^4^ to the Δ^7^ double bond. In Figure S2, the rest of the stacking interactions between the substrate and the enzyme residues are plotted. As can be observed, there are also π–π interactions between Phe352 and the Δ^19^ double bond and His365 and the Δ^10^ double bond. The stacking interaction of DHA with Phe414, which has been described for AA, is established with two double bonds (Δ^13^ and Δ^16^). In the MD replica 1, all those interactions weaken when the PLAT domain separates from the catalytic domain. The principal hALOX12 residues interacting with DHA’s double bonds by stacking interactions are plotted in Figure 7 for the two binding modes observed along the MD replica 1. In replica 2, the stacking interactions with Phe174 remain stable after 80 ns (Figure 6). In Figure S2, it can be observed that the interactions with Phe352 and Phe414 remain very stable all along the trajectory. In contrast, the π–π stacking with His365 is not present in this MD replica.

The PLAT movement also correlates with structural changes in some α-helices of the catalytic domain. In this respect, it is noteworthy that there is a loss of the secondary structure of the α-helix from residue 408 to residue 418, which flips the sidechain of Phe414. With the displacement of the PLAT domain, Phe414 rotates, blocking the U-shaped cavity bottom and opening a new cavity to accommodate the DHA tail. This rotation of the Phe414 sidechain is depicted in Figure 3, Figure 7 and Figure S3. Then, Ile408 approaches the DHA tail that simultaneously rotates around the C_17_–C_18_ single bond, losing the U-shaped binding mode.

**Figure 8:**
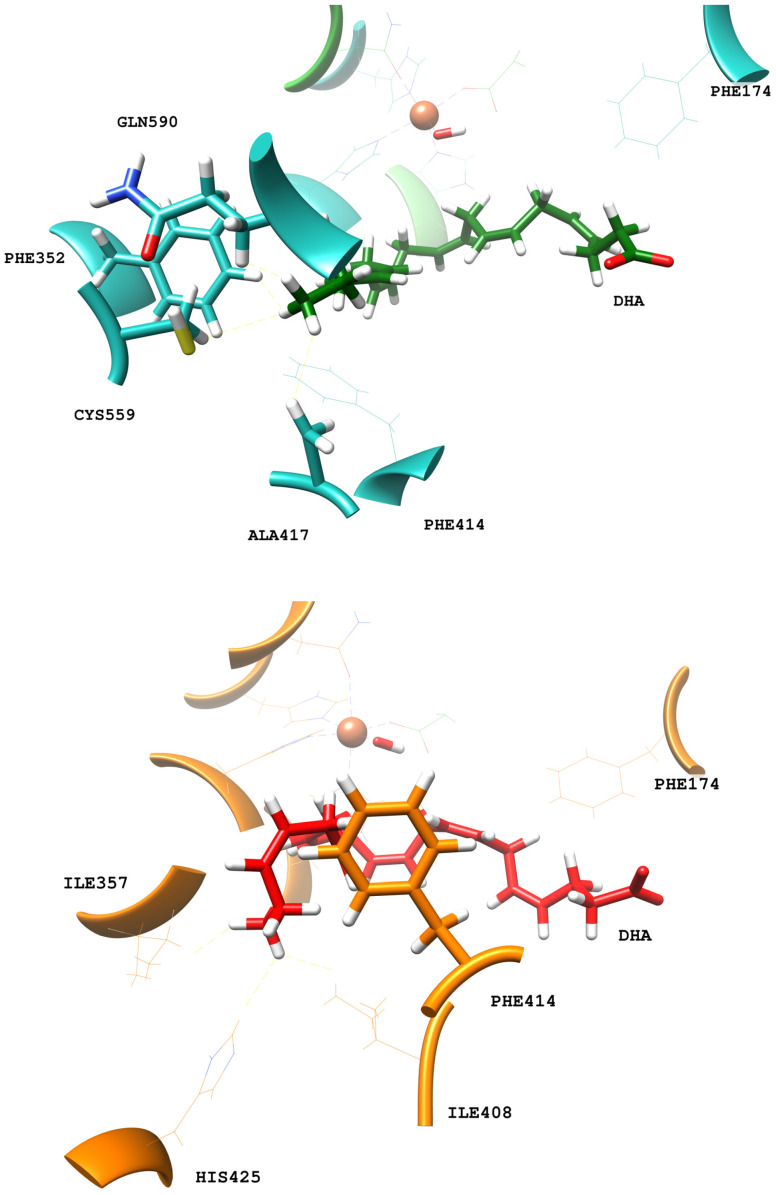
Main interactions between the terminal methyl of DHA and residues of the bottom of hALOX12′s cavity for the close (upper, blue) and open (lower, orange) conformations.

For the U-shaped DHA binding mode, the residues at the cavity bottom that mainly interact with the substrate are Phe352, Ala417, Cys559, and Gln590. For the more twisted DHA orientation in replica 1, the bottom cavity is formed by the sidechains of Ile357, His425, and Ile408. Those residues at the cavity bottom are shown in Figure 8. The changes in the interactions between the substrate’s tail and the protein residues at the cavity bottom could explain why hALOX12 might only partially follow the triad hypothesis. However, kinetic experiments with A417I and V418M mutants would be necessary to confirm this prediction that has already been shown for AA. Finally, it is worth mentioning that Leu407 is also a critical residue defining the U-shaped binding mode of DHA in hALOX12, as has been described for AA [22].

**Figure 9:**
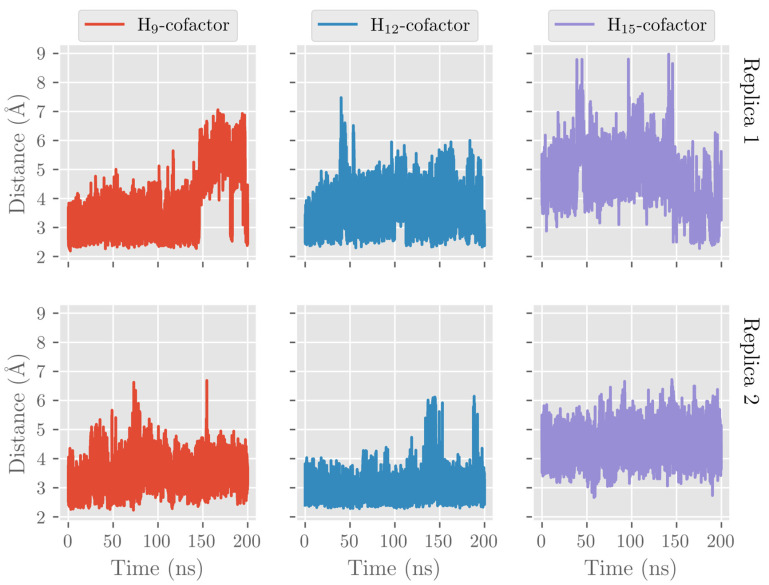
Evolution with time of the H_12_-OH^−^, H_9_-OH^−^, and H_15_-OH^−^ distances along the MD replica 1 and replica 2 in hALOX12.

Here, we also analyzed the evolution of the hydrogen atoms H_9_, H_12_, and H_15_, which are candidates to be abstracted in the first step of the catalytic mechanism. That is, we recorded the distances from the oxygen atom in the Fe(III)-OH^−^ cofactor to the closest H_9_, H_12_, and H_15_ atoms (attached to C_9_, C_12_, and C_15_, respectively) along the MD trajectory (see Figure 9). During the MD replica 1, hydrogen H_12_ remains at precatalytic distances (that is, smaller than 4 Å), although with some structural fluctuations. H_9_ also remains quite stable at precatalytic distances from the cofactor but moves farther away when the PLAT domain separates. The average H_12_-OH^−^ and H_9_-OH^−^ distances before the PLAT movement are 3.60 Å and 3.16 Å, respectively. The smaller average H_9_-OH^−^ distance compared to the H_12_-OH^−^ one does not agree with the regioselectivity of hALOX12, which gives 14-H(p)DHA as the main product. However, we have shown in previous mechanistic studies on the reactivity of other ALOXs that interatomic distances alone cannot explain the molecular origin of enzyme regiospecificity. As can be observed in Figure 9, H_15_ is more distant from the cofactor than H_12_ and H_9_ when the two-domain protein is closed but approaches when the two domains open. The evolution of those three distances indicates that the opening of the two-domain protein is correlated with a movement of DHA to the entrance of the cavity, as mentioned above. Along the MD replica 2, the three distances maintain more stable values in accordance with the stability of the DHA binding mode in this trajectory. In this case, the average H_12_-OH^−^ distance is the smallest (2.99 Å) followed by the average H_9_-OH^−^ distance (3.44 Å), and the H_15_-OH^−^ one (4.49 Å), in agreement with the regiospecificity observed for hALOX12.

**Figure 10:**
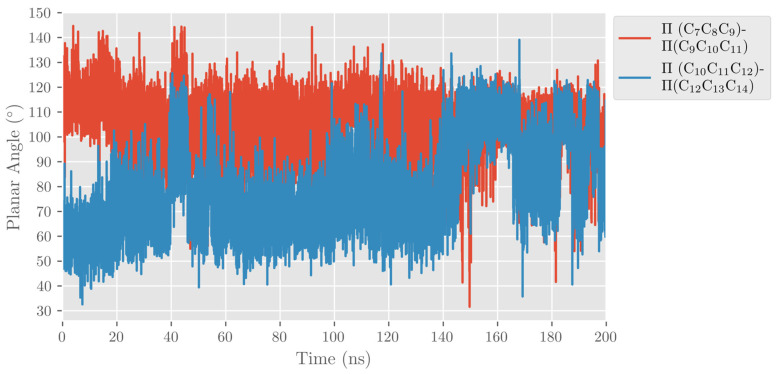
Evolution with time of the angle between planes formed by C_7_C_8_C_9_ and C_9_C_10_C_11_ (in red) and by C_10_C_11_C_12_ and C_12_C_13_C_14_ (in blue) along the MD replica 1 in hALOX12.

As mentioned above, ALOXs are also enzymes that are highly stereospecific. ALOX12 catalyzes the formation of 14S-hydroperoxy-4Z,7Z,10Z,12E,16Z,19Z-docosahexaenoic acid (14S-H(p)DHA), which is the intermediate that forms 13S,14S-epoxy-4Z,7Z,9E,11E,16Z,19Z-docosahexaenoic acid (13S,14S-epoxy-DHA). This epoxide is then enzymatically hydrolyzed to form the final maresin product (MaR1, 7R,14S-dihydroxy-4Z,8E, 10E,12Z,16Z,19Z-docosahexaenoic acid). The stereochemistry of the bonds around C_12_ in 14S-H(p)DHA is critical for the formation of MaR1. We analyzed the MD trajectories of the DHA/hALOX12 complex and concluded that configurations of DHA with the angle between planes Π(C_10_C_11_C_12_) and Π(C_12_C_13_C_14_) smaller than 90° are prompted to give the E stereochemistry of the Δ^12^ bond in 14S-H(p)DHA. Most of the configurations of the MD replica 1 before the PLAT movement accomplish that structural condition, and there is also a fraction of those configurations when the two domains are separated (see Figure 10). In contrast, many DHA configurations present angles between the Π(C_7_C_8_C_9_) and Π(C_9_C_10_C_11_) planes with values above 90°. These configurations would lead to a Z stereochemistry of the Δ^9^ bond in 11S-H(p)DHA.

### 2.2. QM/MM Calculations of DHA Hydroperoxidation Catalyzed by hALOX12

#### 2.2.1. Hydrogen Abstraction Reactions Catalyzed by hALOX12

**Figure 11:**
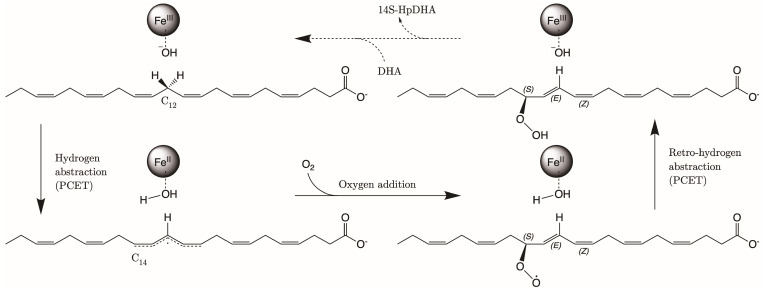
Scheme of the DHA hydroperoxidation mechanism. PCET stands for proton coupled electron transfer.

In Table 1 and Table S1, the QM/MM results corresponding to the first step of the catalytic mechanism are given (see Figure 11). We have calculated the potential energy profiles for the H_12_, H_9_, and H_15_ abstraction reactions using as initial structures several selected snapshots of the MD trajectory for replica 1. Those initial or precatalytic structures were filtered according to the following criteria. We searched precatalytic snapshots in which the initial distance between the three hydrogen atoms, H_12_, H_9,_ and H_15_, and the oxygen atom of the hydrogen acceptor, that is, the oxygen of the Fe(III)-OH^−^ cofactor, was smaller than 4.0 Å.

**Figure 12:**
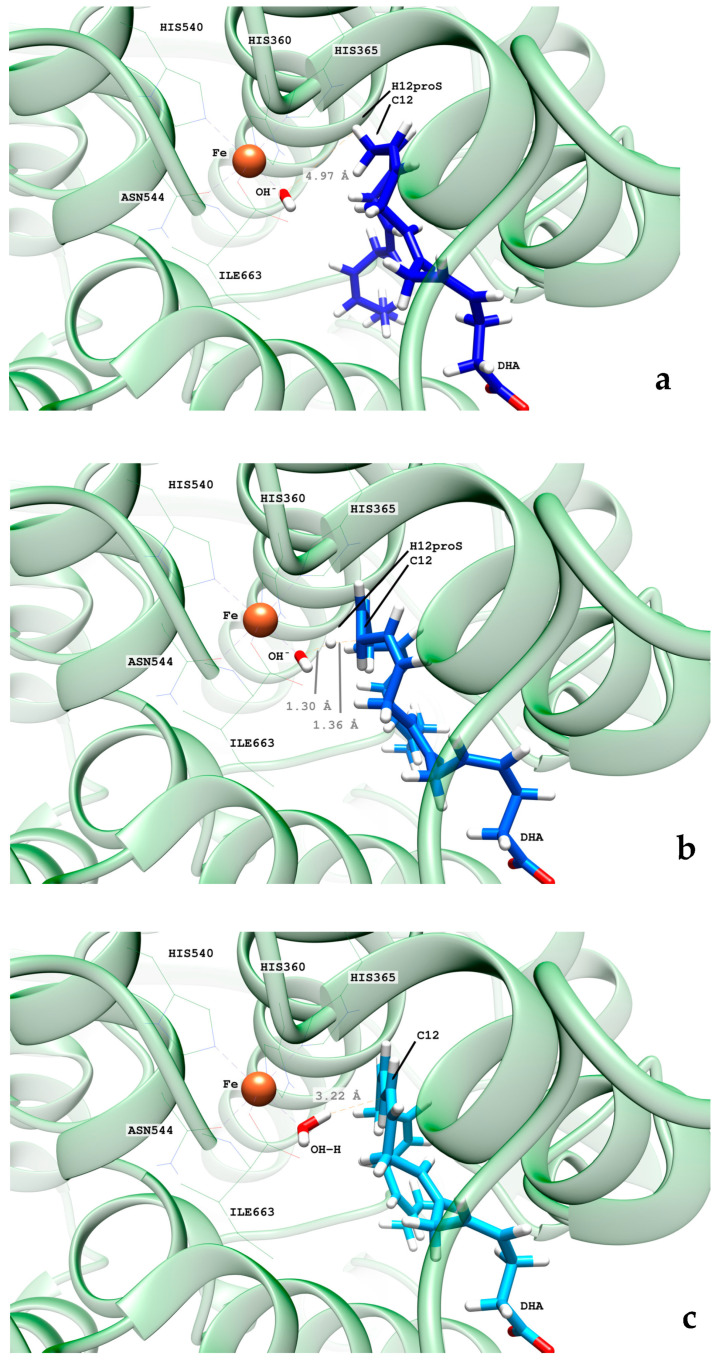
Images of the optimized reactant (**a**), transition state structure (**b**), and product (**c**) of the H_12proS_ abstraction initiated from snapshot 8721 in hALOX12. The distance between H_12proS_ and the oxygen atom in OH^−^ is shown for the optimized reactant (**a**). At the transition state structure, the distances between the shifting hydrogen with respect to C_12_ (donor atom) and the oxygen atom in OH^−^ (acceptor atom) are depicted (**b**). The distance between the H atom in the nascent water molecule and C_12_ is plotted at the optimized product (**c**).

Then, several of those precatalytic snapshots were optimized according to the QM/MM model described in the methodology section, and the corresponding minima were located. From those minima, the potential energy profile for the H-abstraction was calculated using the difference between the C_X_-H_X_ distances and the corresponding H_X_-OH^−^ ones as the reaction coordinate. In Table 1, the results for the most favorable abstraction reactions, the H_12proS_ and H_9proR_ abstractions, are collected. In Table S1, we have included the results for the rest of the H-abstraction energy profiles. The values of the H_proX_-OH^−^ distances in Table 1 are given for the optimized reactants of the H_12_ and H_9_ abstraction processes. As can be observed, the QM/MM optimized distances are longer than the initial distances of the precatalytic structures. As for the potential energy barriers, there is no correlation between their values and those of the optimized H_12proS_-OH^−^ or H_9proR_-OH^−^ distances. Thus, the H_9proR_-OH^−^ distances are shorter in almost all the optimized reactants, but the individual barriers are higher for the H_9_-abstraction than for the H_12_-abstraction. At the optimized radical products of both H-abstractions, the corresponding pentadienyl group is planar, and the electronic density is delocalized over five carbon atoms. In DHA, the set of carbon atoms around C_12_ that will form the pentadienyl radical after H_12_-abstraction is closer to planarity than the set of carbon atoms around C_9_, which will form the pentadienyl radical after H_9_-abstraction. The larger the geometrical change from an initially nonplanar structure to a planar pentadienyl and, especially, the greater steric hindrance to that motion, the higher the contribution to the potential energy barrier [24]. Both abstraction processes are exoergic, as has already been obtained in other ALOXs with AA. All in all, the exponential average energy barrier is 6.8 kcal/mol lower for H_12_-abstraction than for H_9_-abstraction. This result agrees with the regioselectivity of hALOX12, which favors the abstraction of H_12_, leading to the formation of 14S-H(p)DHA after the addition of O_2_ at C_14_ (see next section). In Table 1, the pentadienyl stereochemistry is given at both product radicals. It is remarkable that the stereochemistry of all the pentadienyl radicals from C_10_ to C_14_ (ZE) is the one needed for leading to the fully characterized 14S-hydroperoxo-4Z,7Z,10Z,12E,16Z,19Z-docosahexaenoic acid (see next section). (ZE) stands for the stereochemistry in the pentadienyl radical that will become 10Z, and 12E in 14S-H(p)DHA. Hence, the geometry of the QM/MM optimized products ratifies the analysis made of the most stable configurations of DHA along the MD trajectory (See Figure 10). In contrast, the stereochemistry of the pentadienyl radical centered at C_9_ is (ZZ). In this case, the stereochemistry of 11-H(p)DHA, being the minor product, has not been reported. In Figure 12, the structures of the optimized reactant, transition state structure, and product of the H_12proS_-abstraction initiated from snapshot 8721 are depicted.

On the other hand, the H_15_-abstraction (Table S1) has a low probability of occurring since the percentage of precatalytic structures (10%, see Figure 9) is quite lower than for the H_12_ (74%) and H_9_ (73%) abstractions.

#### 2.2.2. O_2_ Addition and Retro-Hydrogen Abstraction Reactions Catalyzed by hALOX12

The second step of the overall DHA hydroperoxidation process that leads to the final products consists of adding an oxygen molecule to the C_10_–C_14_ or C_7_–C_11_ DHA pentadienyl radicals, formed once H_12_ or H_9_ has been abstracted, respectively. Here, we only calculated the reaction pathway for O_2_ addition to the delocalized C_10_–C_14_ radical that gives the main hydroperoxide product, 14S-H(p)DHA.

**Figure 13:**
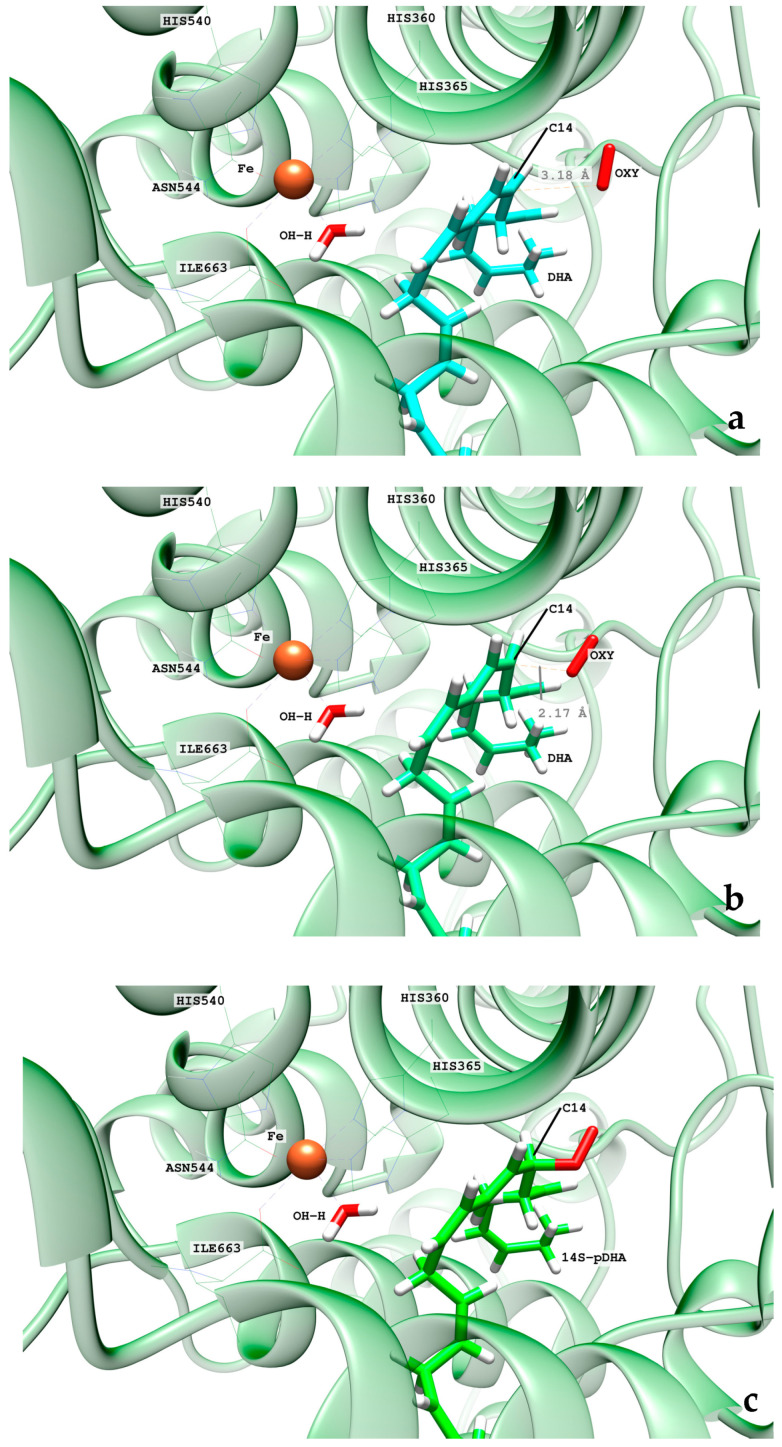
Images of the optimized reactant (**a**), transition state structure (**b**), and product (**c**) of the O_2_ addition at C_14_ (frame 8721) in hALOX12. The distances between C_14_ and the attacking oxygen atom are given for the reactant and transition state structures.

We have explored different initial locations for the O_2_ molecule around C_14_ at the conformation of the DHA pentadienyl radical obtained from the different MD snapshots. The O_2_ molecules around C_14_, taken as the origin of coordinates, have been initially placed at 3.0 Å along the x, y, and z Cartesian axes and along the bisector axes contained in the xy, xz, and yz planes. The structures selected were chosen by a visual analysis. This means that all the O_2_ molecules with close contacts or clashes with other residues were discarded. Then, QM/MM single-point energy calculations were carried out for the selected positions, and the higher energy structures were discarded. The most stable structures were optimized and then taken as starting points to build the reaction path for the oxygen addition to C_14_. The addition pathway was calculated in the forward and backward directions using the distance from the attacking oxygen of the O_2_ molecule to C_14_ as the reaction coordinate. From the last structure of the backward path, the addition reactant structure was optimized. The O-C_14_ distance values for the converged QM/MM minimum energy geometries are presented in Table 2. All the oxygen addition reaction pathways present low potential energy barriers. The chirality of the oxygenated product and the geometry of the addition pathway are also included in Table 2. We have obtained three converged addition pathways corresponding to antarafacial additions leading to peroxyl radicals with S stereochemistry at C_14_, in agreement with the experimental stereochemistry assigned to the final hydroperoxide product, 14S-H(p)DHA. In Figure 13, the reactant, transition state structure, and the product of the addition process to C_14_, initiated from frame 8721, are depicted.

**Figure 14:**
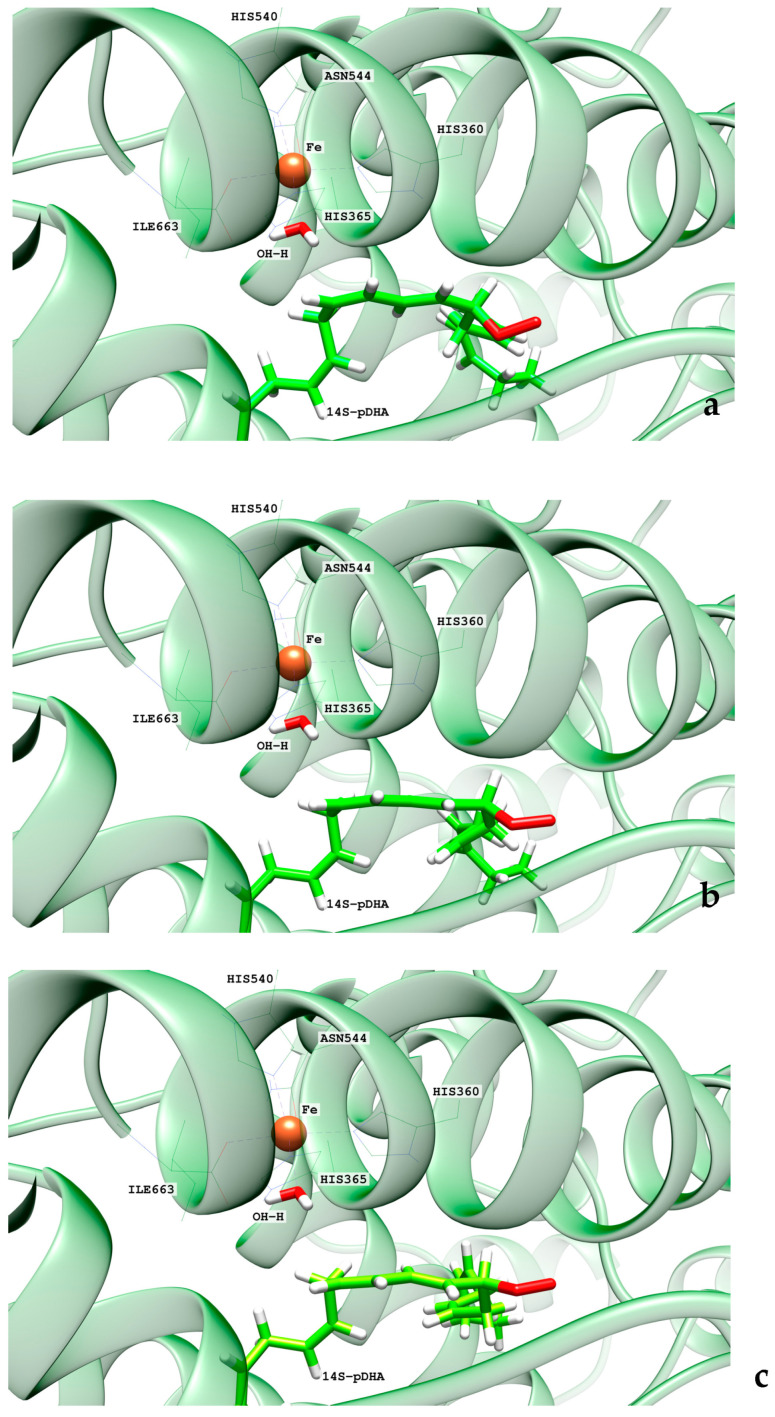
Images of the optimized reactant (**a**), transition state structure (**b**), and product (**c**) of the carbon chain rotation of the peroxyl radical (frame 8721) in hALOX12.

The final step of the overall hydroperoxidation mechanism consists of a retro-hydrogen abstraction from the Fe(II)-H_2_O cofactor to the peroxyl DHA radical. This reaction leads to the final 14S-H(p)DHA product, and hALOX12 recovers the initial Fe(III)-OH^−^ cofactor’s state. However, as the oxygen molecule has been added following an antarafacial approach with respect to the cofactor, the carbon chain of the peroxyl radical needs to rotate before the proper retro-hydrogen transfer can take place. We calculated the potential energy profiles for this carbon chain rotation using the C_13_C_14_C_15_C_16_ dihedral as the reaction coordinate for the three pathways initiated at snapshots 8721, 10,106, and 18,168. The potential energy barriers measured from the corresponding previous minima (that is, the addition products) on the QM/MM potential energy surface for the three rotations range from 5.8 to 6.6 kcal/mol. In Figure 14, the reactant, transition state structure, and the product of the rotation process, initiated from frame 8721, are presented. Note that the reactant minimum in Figure 14a has the same structure as the addition product minimum in Figure 13c but is plotted from a different perspective to better follow the carbon chain rotation.

**Figure 15:**
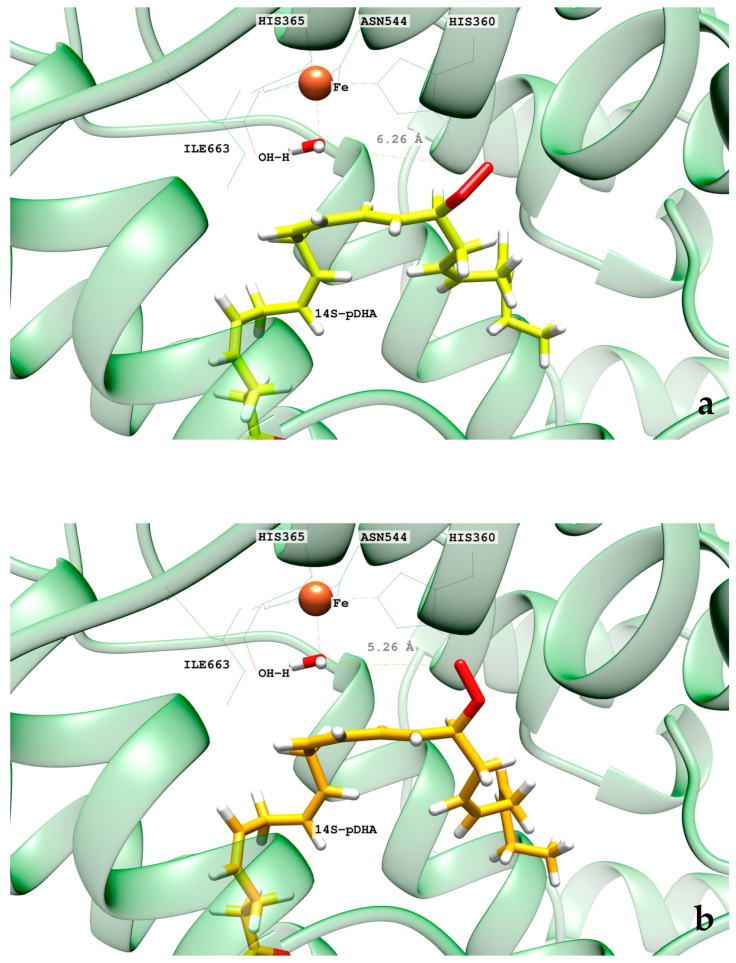

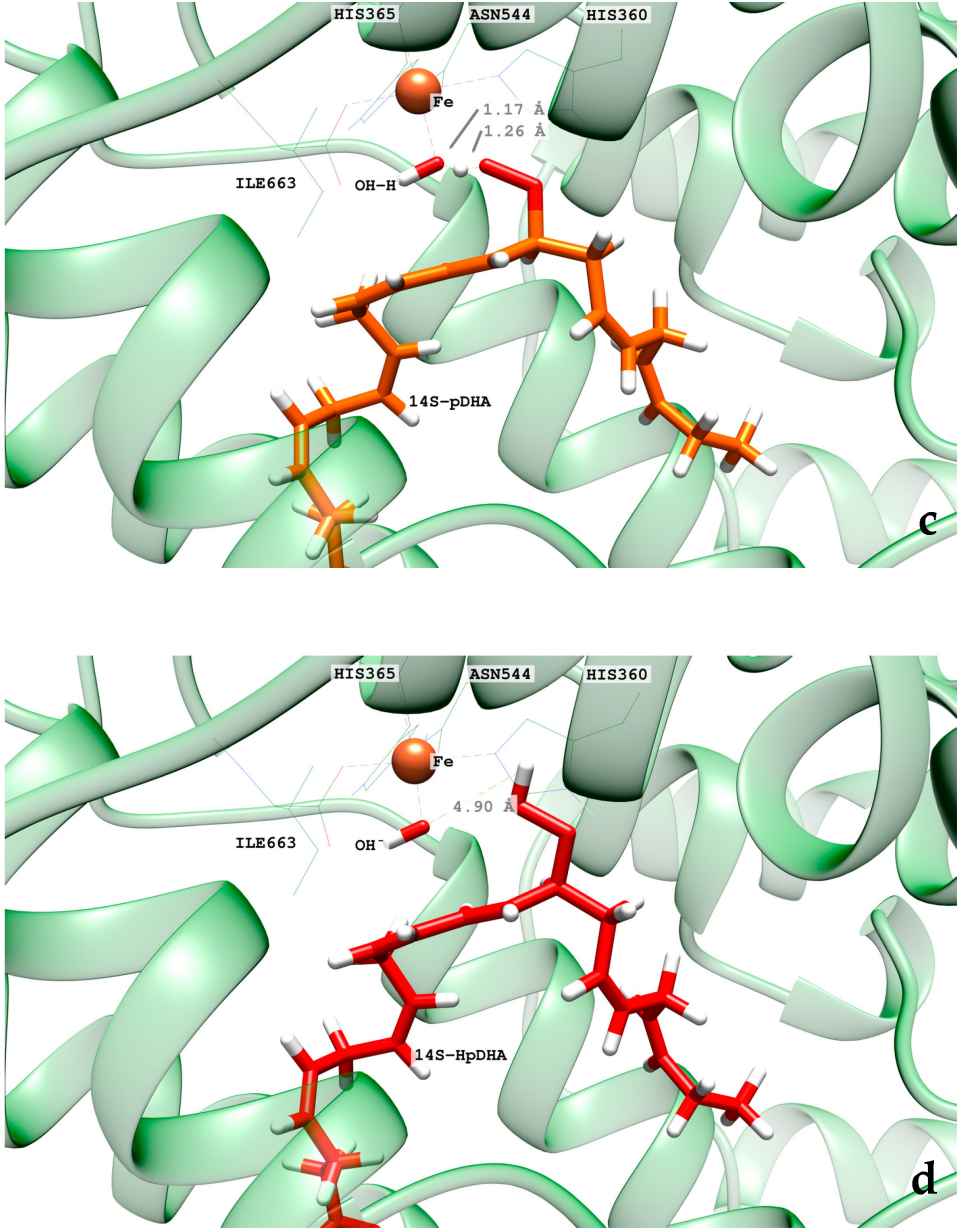
Images of the transition state structure (TS1) (**a**), intermediate (INT1) (**b**), transition state structure (TS2) (**c**), and the final 14S-H(p)DHA product (**d**) (frame 8721) in hALOX12. The distances between the outer oxygen of the peroxo group and the water hydrogen atom are given for TS1 and INT1. The distances corresponding to the shifting hydrogen atom are indicated for TS2 and the final 14S-H(p)DHA product.

From the rotated peroxyl radical the retro-hydrogen abstraction from the Fe(II)-H_2_O cofactor to the peroxyl DHA radical can be initiated. We calculated the corresponding QM/MM potential energy profile versus a reaction coordinate defined as the difference between the breaking H-OH (water ligand) bond distance and the forming H-OOC_14_ one. This abstraction process involves two different structural steps. First, there is a reorganization of the peroxyl radical that correlates with the rotation of the peroxo group at C_14_ from the antarafacial to the suprafacial side. The potential energy barriers given in Table 2 for this structural reorganization (10.2 and 13.1 kcal/mol for the pathways initiated from snapshots 8721 and 18,168, respectively) are calculated from the corresponding reactant minima, which are the products obtained from the rotation pathway. The reorganization corresponding to snapshot 10,106 did not converge. The transition state structure (TS1) and the product geometry or intermediate (INT1) of this reorganization process are depicted in Figure 15 for the reaction initiated from snapshot 8721. The final step of the mechanism corresponds to the movement of the shifting hydrogen. The abstraction potential energy barriers calculated from the optimized intermediate are 33.3 and 20.6 kcal/mol for the pathways initiated from frames 8721 and 18,168, respectively. In Figure 15, the plots of the stationary-point structures (transition state (TS2) and product) of this final step are also depicted for the pathway of snapshot 8721.

**Figure 16:**
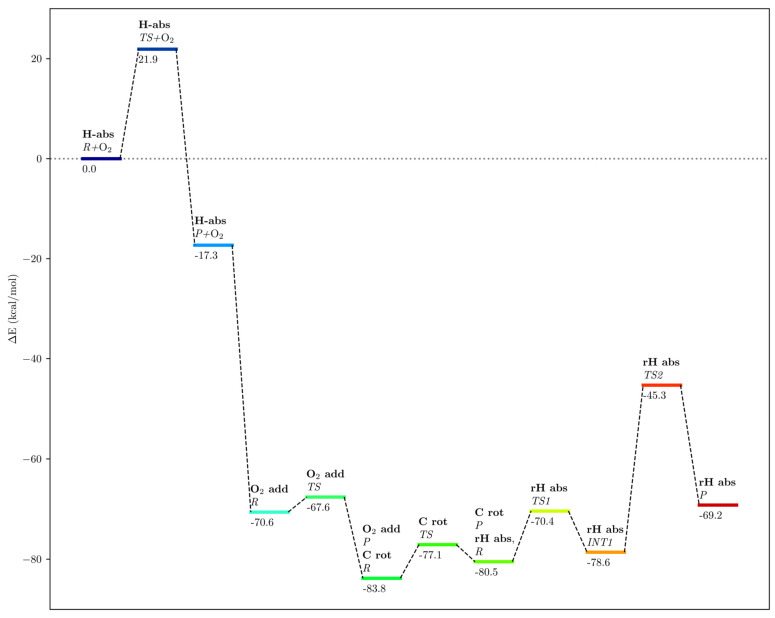
Overall energy scheme of the H_12proS_-abstraction, oxygen addition, carbon chain rotation, and retro-hydrogen abstraction steps of the DHA hydroperoxidation mechanism in hALOX12. All energies are in kcal/mol. The zero of energies corresponds to the reactant of the H_12proS_ abstraction step bound to the solvated enzyme plus an oxygen molecule within the water box whose position has been optimized at 12.1 Å from the substrate’s C_14_.

In Figure 16, we have depicted an overall energy scheme of the mechanistic steps initiated from the reactant of the H_12proS_-abstraction reaction (frame 8721) bound to the solvated enzyme plus an oxygen molecule within the water box at the entrance of the enzyme cavity. The position of the oxygen molecule has been optimized at 12.1 Å from the C_14_ atom of the substrate. This structure has been taken as the zero of energy of the overall energy scheme. The approach of the oxygen molecule to 3.1 Å from C_14_ at the addition reactant structure represents a falloff in energy of 53.3 kcal/mol with respect to the H_12proS_-abstraction product. Consequently, the barriers for the O_2_ addition, carbon chain rotation, and retro-hydrogen abstraction are very far below the reference structure. Therefore, none of those processes can be the rate-determining step of the hydroperoxidation reaction. In contrast, the H_12proS_-abstraction transition state structure is 17.3 kcal/mol above the reference structure (see Table 1 and Figure 16), so it is the rate-determining step. Here, we are assuming that the oxygen molecule approaches the enzyme once the H-abstraction process has taken place.

### 2.3. MD Simulations of the DHA/pigALOX15-mini-LOX Complex

In this section, the results corresponding to the two MD trajectories of the solvated DHA/pigALOX15-mini-LOX complex are presented for the sake of comparison with DHA/hALOX12 behavior. In Figure S4, we have plotted the protein backbone RMSD versus time for the two MD replicas. The results show a stable conformation of the C-terminal domain of pigALOX15-mini-LOX, even though the N-terminal domain is not present. The carbon-chain RMSD of DHA also indicates a rather steady binding mode along the two trajectories.

**Figure 17:**
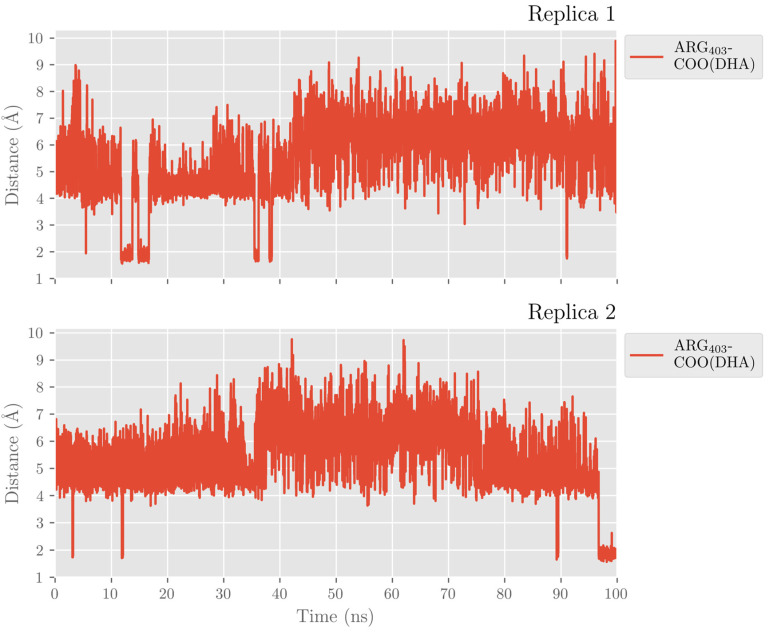
Distances of a carboxylate’s oxygen atom of DHA to the closest hydrogen atom in Arg403 versus time for the MD replica 1 and replica 2 in pigALOX15-mini-LOX.

In the pigALOX15-mini-LOX active site, DHA also adopts a U-shaped binding mode like that described in hALOX12. However, the carboxylate head of DHA does not interact with Arg403 (Arg402 in hALOX12), as can be observed in Figure 17 along the two MD trajectories.

**Figure 18:**
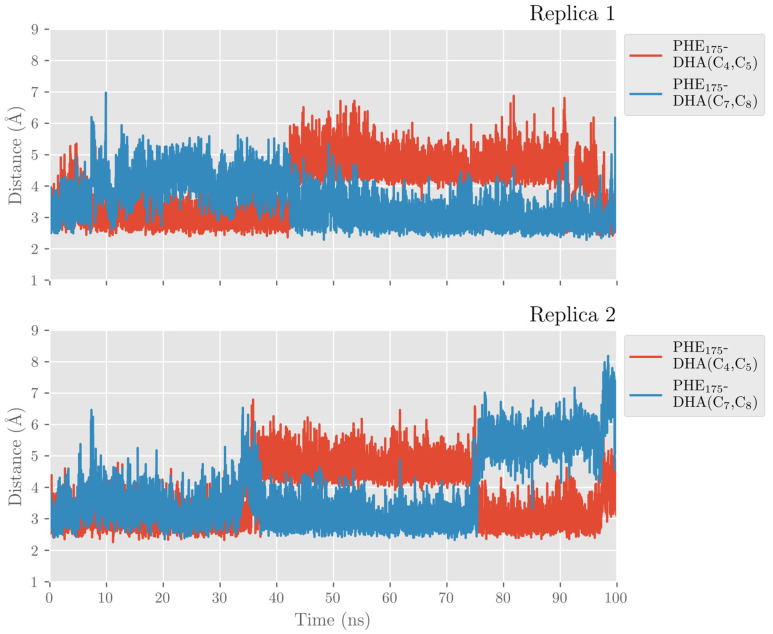
Distances between Phe175 sidechain and the closest atom of the Δ^4^ and Δ^7^ double bonds versus time for the MD replica 1 and for replica 2 in pigALOX15-mini-LOX.

This interaction is lost because a displacement of the α2-helix in pigALOX15-mini-LOX moves away from the Glu176…Arg403 pair (Glu175…Arg402 in hALOX12) from the cavity entrance. Moreover, pigALOX15-mini-LOX has a Gly in position 407 instead of the Gln406 of hALOX12, which does not interact with Arg403 and cannot maintain this arginine close to the DHA carboxylate. Therefore, in its location in pigALOX15-mini-LOX, the carboxylate head of DHA only interacts with some water molecules. In addition, DHA also establishes π–π interactions between its double bonds and aromatic residues of the active site that stabilize the substrate binding mode. The stacking interactions between Phe175 and Δ^4^ and Δ^7^ are plotted in Figure 18. Along the MD replicas, Phe175 exchanges its interaction between those two double bonds, except during the first 30 ns of replica 2 when the stacking is present with both double bonds at the same time. The rest of the π–π interactions presented in Figure S5 are weaker in pigALOX15-mini-LOX than in hALOX12.

**Figure 19:**
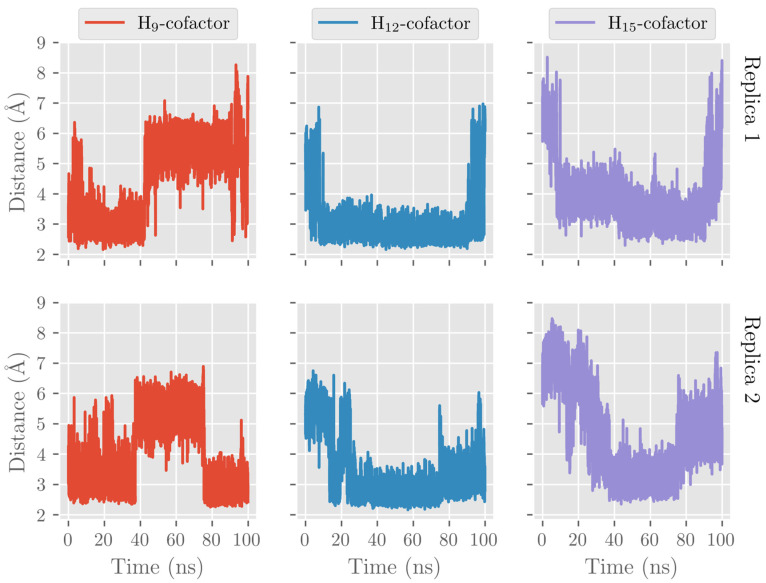
Evolution with time of the H_12_-OH^−^, H_9_-OH^−^, and H_15_-OH^−^ distances along the MD replica 1 and replica 2 in pigALOX15-mini-LOX.

As for H_12_-OH^−^, H_9_-OH^−^, and H_15_-OH^−^ distances along the MD replica 1 and replica 2, we have plotted them in Figure 19. H_12_ remains the closest to the OH- group of the cofactor the main part of the trajectory. H_9_ and H_15_ present a similar number of precatalytic structures when considering both MD replicas. However, when H_9_ is closer, H_15_ is farther away, and vice versa. Our MD simulation indicates that the DHA binding mode in pigALOX15-mini-LOX could lead to 14-H(p)DHA as a major product but also, with less probability, to 11-H(p)DHA and 17-H(p)DHA as minor products. Experimentally, Kühn and coworkers obtained a relative share of 76.4% for 14-H(p)DHA and 23.6% for 17-H(p)DHA for the specificity of pigALOX15 [21].

**Figure 20:**
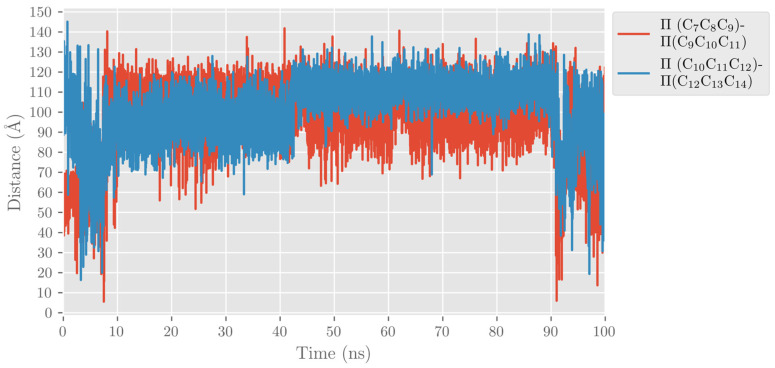
Evolution with time of the angle between planes formed by C_7_C_8_C_9_ and C_9_C_10_C_11_ (in red) and by C_10_C_11_C_12_ and C_12_C_13_C_14_ (in blue) along the MD replica 1 in pigALOX15-mini-LOX.

When we calculated the angle between planes Π(C_10_C_11_C_12_) and Π(C_12_C_13_C_14_) of DHA, the values obtained are above 90° most of the time (see Figure 20). As indicated before, this result means that the configurations of DHA would give a Z stereochemistry of the Δ^12^ bond in 14S-H(p)DHA. The angle between planes Π(C_7_C_8_C_9_) and Π(C_9_C_10_C_11_) of DHA behaves similarly all along the trajectory.

### 2.4. QM/MM Calculations of H_12_-Abstraction from DHA Catalyzed by pigALOX15-mini-LOX

In this section, we present the results of the QM/MM calculations corresponding to the H_12_-abstraction. We have calculated the potential energy profiles for the H_12_ transfer using as initial structures several selected snapshots of the MD trajectory for replica 1. Following the methodology explained in the corresponding section, we calculated the potential energy profile of this catalytic step using, as a reaction coordinate, the difference between the C_12_-H_12_ and the H_12_-OH^−^ distances. In Table 3, the potential energy barriers are given along with their exponential average. The obtained value of 17.6 kcal/mol is nearly the same as for the H_12proS_-abstraction in hALOX12. This theoretical result verifies, in agreement with experiments, that pigALOX15-mini-LOX is active and can catalyze the conversion of DHA to 14-H(p)DHA beginning with the abstraction of H_12proS_ [21]. In Table 3 the stereochemistry of the pentadienyl radical centered at C_12_ is given. All the radical products present a (ZZ) stereochemistry, as the configurations of the MD trajectory had predicted. This is not such a stable stereochemistry for those pentadienyl radicals in the enzyme’s binding pocket as the (ZE) configuration obtained in hALOX12. Hence, the corresponding reaction energies are not as negative as in hALOX12 (Table 1). Moreover, as commented above, the (ZZ) stereochemistry is not the adequate configuration for the formation of 13S,14S-epoxy-4Z,7Z,9E,11E,16Z,19Z-docosahexaenoic acid and for finally leading to 7R,14S-dihydroxy-4Z,8E, 10E,12Z,16Z,19Z-docosahexaenoic acid (MaR1). For this reason, our hypothesis here is that pigALOX15-mini-LOX forms 14S-hydroperoxo-4Z,7Z,10Z,12Z,16Z,19Z-docosahexaenoic acid, but this hydroperoxide would not give the bioactive SPM MaR1 that it is synthesized by hALOX12.

## 3. Materials and Methods

### 3.1. Protein Setup

The model structure of *human* ALOX12 was taken from the AlphaFold Data Base (UniprotID P18054) [42]. For pigALOX15-mini-LOX, we used the x-ray structure launched at the Protein Data Bank with PDB code 3RDE [36], removing the ligand bound to the protein. The two structures were protonated through a web interface (www.playmolecule.org) (accessed on 19 January 2021) [47]. A pH = 7.0 was employed for the titratable residues. The protonation state for the iron coordination sphere was corrected by hand to ensure a correct description.

### 3.2. Molecular Docking Simulations

Docking calculations were performed with GOLD5.8 [48] to obtain stable binding modes of DHA in the active site of hALOX12 and pigALOX15-mini-LOX. For the conformational search of the ligand, the torsion angle distribution of the Cambridge Database was used. In contrast, some protein side chains were treated as flexible. The GOLD’s option to consider the interactions of organic ligands with metal ions in metalloenzymes was activated, but it restricted the docking exploration to hexacoordinated geometries of iron. The binding side was defined as a sphere of 20 Å around the iron atom. This conformational space was explored using the Lamarckian algorithm included in GOLD. One hundred DHA-binding poses were generated and grouped into clusters. We used the ChemScore fitness function to rank the docking poses and estimate their binding free energies.

### 3.3. Molecular Dynamics Simulations

The best-ranked docking poses of DHA bound to hALOX12 and pigALOX15-mini-LOX were selected to initiate the MD simulations. For the solvated DHA/hALOX12 complex, we used the ff19SB force field [49] to calculate the potential energy of the protein and the OPC force field [50] to describe the water molecules, as recommended [49]. For the solvated DHA/pigALOX15-mini-LOX complex, we used the ff14SB force field [51] for the protein and the TIP3P force field to describe the water molecules as recommended [51]. We developed specific parameters for DHA using the AMBER standard protocol with Antechamber and Parmchk2 modules. The GAFF2 library [52] was used as the source for these parameters. The substrate structure was optimized employing the B3LYP/6-31G(d) level of theory. The atomic charges were set to fit the electrostatic potential generated at the B3LYP/6-31G(d) level of theory by the restrained electrostatic potential (RESP) model. The atomic charges were calculated according to the Merz–Kollman scheme [53] using Gaussian16 [54]. An unprotonated state was established for the DHA substrate in the two complexes, considering the physiological conditions. We also developed specific MM parameters for the iron atom and its first coordination sphere using the MCPB.py procedure [55] within the bonded model and the Seminario method for the force constant calculations [56]. For hALOX12, the iron ligands are His360, His365, His540, Asn544, Ile663, and the OH^−^ group. For pigALOX15-mini-LOX, the iron ligands are His250, His255, His430, His434, Ile552, and the OH^−^ group.

The protocol recommended by the AMBER package using the tLeap module was used to assemble the DHA/hALOX12 and DHA/pigALOX15-mini-LOX systems, solvate those complexes with an orthorhombic box of pre-equilibrated water molecules with a buffer of 10 Å and neutralize the total charge by adding sodium cations. The resulting systems contain around 80,000 atoms, of which about 10,600 belong to the protein, for the DHA/hALOX12 system, and 64,000 atoms, of which about 8800 belong to the protein, for the DHA/pigALOX15-mini-LOX. The rest of the atoms correspond to water molecules and salt ions.

The molecular dynamics (MD) simulations were run using either the AMBER20 (hALOX12) [57] or AMBER16 (pigALOX15-mini-LOX) [58] GPU (CUDA) version of the PMEMD package [59,60]. Initially, the systems were submitted to 110,000 energy minimization steps combining the steepest decent and conjugate gradient methods to remove close contacts. In the first 5000 steps, harmonic restraints were applied to the protein and substrate atoms with a force constant of 5.0 kcal mol^−1^ Å^−2^ so that only the solvent and ions were relaxed. In the following 5000 steps, harmonic restraints were applied to the protein backbone with the same force constant as before. Finally, the whole system was kept free during the last 100,000 steps. Then, MD simulations using periodic boundary conditions and the particle-mesh Ewald approach to introduce long-range electrostatic effects were performed. The DHA/hALOX12 system was gently heated using six 20 ps steps, incrementing the temperature by 50 K each step (0–300 K) under constant volume. For the DHA/pigALOX15-mini-LOX system, the temperature was increased by 10 steps of 30 K (0–300 K) during 20 ps each step. After heating, we calculated an MD trajectory of 1 ns within the NPT ensemble (300 K, 1 atm) to adjust the volume of the orthorhombic box and relax the density to a value of around 1 g cm^−3^. During the heating and the compressing, harmonic restraints were applied to the protein backbone with a force constant of 5.0 kcal mol^−1^ Å^−2^, whereas the rest of the system was kept free. The Langevin equilibration scheme [61] was used to control and equalize the temperature, while the pressure was adjusted by the Berendsen barostat [62]. Next, an equilibration stage of 10 ns at constant volume and temperature (300 K) was performed. Finally, we ran production MD trajectories of 250 ns and 100 ns for hALOX12 and pigALOX15-mini-LOX, respectively, within the same NVT ensemble. For each system, two MD replicas were calculated. A time step of 1 fs was used along the whole MD trajectories. Bonds involving hydrogen were constrained with the SHAKE algorithm [63]. The non-bonding interactions have been calculated with a cutoff of 9 Å.

The analysis of the MD trajectories was carried out with a Python package and RCBS.py [64]. Matplotlib was used for plotting [65].

### 3.4. QM/MM Calculations

The modular program package ChemShell 3.7 [66,67] was employed to carry out the QM/MM computations. TURBOMOLE 7.0 [68] was used for the DFT calculations and the DL_POLY 5.0 [69] module in ChemShell for the MM part.

**Figure 21:**
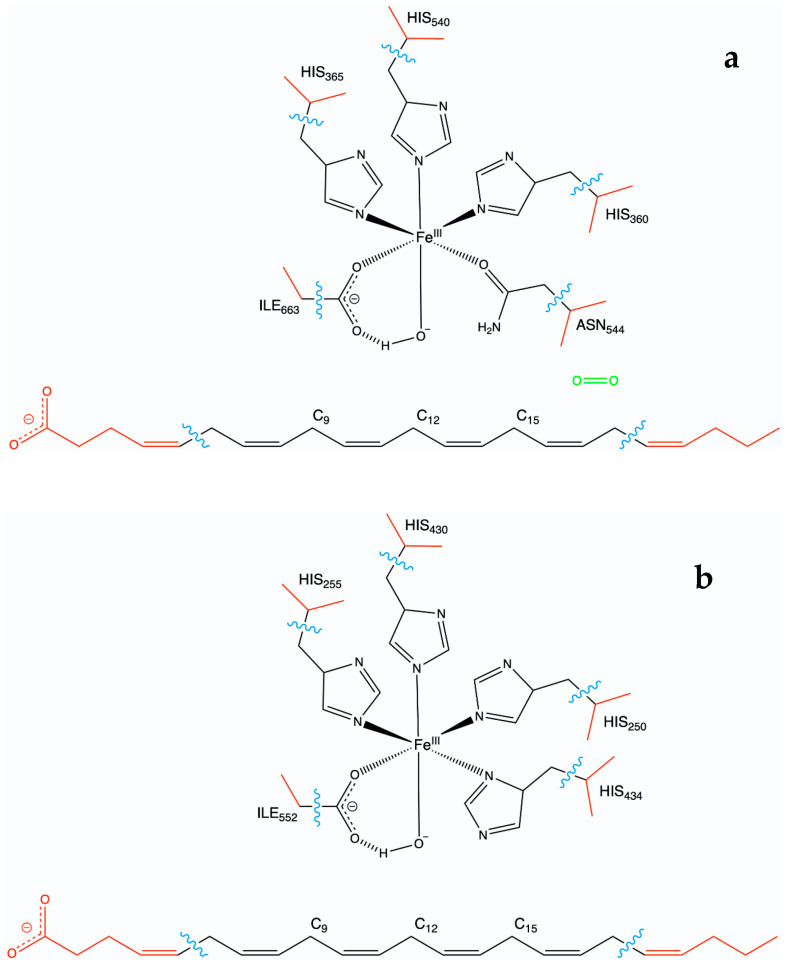
QM/MM partition in the DHA/hALOX12 (**a**) and DHA/pigALOX15-mini-LOX systems (**b**).

The QM region was described by the B3LYP hybrid function [70]. The 6–31G(d) Pople basis set [71] was employed for the C, H, O, and N atoms, while the LANL2DZ basis set [72] was used for the Fe atom. The QM/MM partition is depicted in Figure 21. As for the hydrogen abstractions, the QM region was defined by all the atoms of the DHA substrate, which are found between C_6_ and C_17_; 11 atoms for each His residue of the iron coordination sphere (His360, His365, and His540 in hALOX12 and His250, His255 His430, and His434 in pigALOX15-mini-LOX); 8 atoms of the iron ligand Asn554 in hALOX12; and 3 atoms of the iron ligand Ile residue (Ile 663 in hALOX12 and Ile552 in pigALOX15-mini-LOX) and the Fe^III^-OH^−^ cofactor. For oxygenations, the DHA carbon chain rotation, and the hydrogen retrodonation, this region was enlarged by an oxygen molecule. Seven link atoms were included to define the QM/MM boundary: five between the bonds Cα-QM atoms of the five residues in the iron coordination sphere and two bonded to the aliphatic carbon atoms of the lipid substrate (placed between C_5_-C_6_ and C_17_-C_18_). An electronic embedding scheme was employed to treat the interaction between the QM and MM subsystems. We also used the charge shift algorithm to minimize overpolarization effects. Cutoffs were not introduced to treat the nonbonding MM and QM/MM interactions [73].

The QM/MM optimizations have been carried out by employing the limited-memory Broyden–Fletcher–Goldfarb–Shanno (L-BFGS) algorithm [74] for energy minimizations and scans of reaction pathways. For these optimizations to minima, a microiterative scheme [75] was considered using hybrid delocalized coordinates (HDLC) [76]. As for the transition-state searches, the dimer method was used [77]. These algorithms are implemented in the DL_FIND geometry optimization library [78] of ChemShell.

For these QM/MM models, all water molecules outside a 17 Å radius volume centered on the ligand molecule were removed. The active region was defined by all residues and water molecules inside a 15 Å radius sphere centered on C_12_ of the ligand molecule. This region was allowed to move freely (≈2200 atoms), while the atoms left out were kept frozen during the optimization. Roughly 12,000 atoms were considered in the QM/MM calculations.

The images of structures were plotted with UCSF CHIMERA [79].

## 4. Conclusions

*Human* ALOX12 is a lipoxygenase that catalyzes the oxidation of the ω-3 fatty acid 4,7,10,13,16,19-docosahexaenoic acid (DHA), forming the hydroperoxides, 14S-H(p)DHA and 11S-H(p)DHA, as major and minor products, respectively. The biological relevance of the corresponding reduced oxylipins, 14S-hydroxydocosahexaenoic acid (14S-HDHA) and 11S-hydroxydocosahexaenoic acid (11S-HDHA), has been associated with the regulation of thrombus formation in vivo. PigALOX15 also catalyzes the conversion of DHA to 14S-H(p)DHA, but the minor product detected is 17S-H(p)DHA.

In this paper, we have carried out docking and molecular dynamics simulations, plus QM/MM calculations, to analyze the molecular details of the complete DHA hydroperoxidation mechanism in hALOX12. For the first time, the protein setup of hALOX12 has been modeled using the structure provided by the AlphaFold server. For pigALOX15, the same methodology has been used to calculate the energy barrier of the first step of the catalytic mechanism using the X-ray structure of an N-terminal truncation variant of pigALOX15.

The DHA peroxidation reaction is initiated by the H-abstraction of H_12,_ H_9_, or H_15_ of DHA by the Fe (III)-OH^−^ cofactor. Next, an oxygen molecule inserts on C_14_, C_11_, or C_17_ following an antarafacial approach. Finally, there is a retro-hydrogen donation from the Fe (II)-H_2_O cofactor to the peroxyl radical, leading to the corresponding hydroperoxides with S stereochemistry. The calculated QM/MM energy barriers for each mechanistic step confirm that the reaction process is viable in the hALOX12 active site. Interestingly, the stereochemistry (ZE) of the pentadienyl radicals from C_10_ to C_14_, formed after H_12pros_ abstraction, is the one needed for leading to the fully characterized 14S-hydroperoxy-4Z,7Z,10Z,12E,16Z,19Z-docosahexaenoic acid because (ZE) in the pentadienyl radical becomes 10Z and 12E in 14S-H(p)DHA. Only with this stereochemistry can 14S-H(p)DHA be converted by ALOX12 to 7R,14S-dihydroxy-4Z,8E,10E,12Z,16Z,19Z-docosahexaenoic acid (MaR1). This fact is relevant because MaR1 is a potent SPM that actively participates in the inflammation-resolution phase of many diseases and has also been described as a novel antiplatelet agent.

In pigALOX15-mini-LOX, our calculations support that 14S-H(p)DHA can be formed, but the stereochemistry (ZZ) of the pentadienyl radicals from C_10_ to C_14_, obtained after H_12pros_ abstraction, is not adequate for MaR1 formation.

This is an excellent example of how an enzyme governs the stereochemistry of the catalytic mechanism that leads to a given bioactive product (MaR1 in this case) and how this stereochemistry control might be species specific.

## Figures and Tables

**Table 1:** Number of the MD frame used as initial coordinates for the QM/MM optimization in hALOX12, optimized H_12proS_-OH^−^ and H_9proR_-OH^−^ distances (in Å) at reactants, potential energy barriers, and reaction energies (in kcal/mol) for the H_12proS_ and H_9proR_ abstraction processes. The stereochemistry of the pentadienyl radicals centered at C_12_ and C_9_ is also given. Exponential average (in kcal/mol) energy barriers [46] for the two H-abstractions are included in the last row.

	H_12proS_				H_9proR_			
Frame	${d_{H-OH^{-}}^{react}\;}^{a}$	$\Delta E^{‡}$	ΔE	Pentadienyl Stereochemistry	${d_{H-{OH}^{-}}^{react}\;}^{b}$	$\Delta E^{‡}$	ΔE	Pentadienyl Stereochemistry
**322**	3.5	15.9	−18.3	ZE	3.5	36.4	−13.9	ZZ
**2253**	5.4	20.5	−19.0	ZE	3.5	23.4	−19.1	ZZ
**4986**	5.4	19.3	−17.0	ZE	5.0	35.0	−14.1	ZZ
**6993**	3.9	18.0	−18.5	ZE	3.1	29.7	−14.5	ZZ
**8721**	5.0	21.9	−17.3	ZE	3.3	22.7	−13.6	ZZ
**10,106**	5.5	24.3	−16.8	ZE	3.4	25.6	−17.4	ZZ
**12,860**	5.2	22.9	−18.5	ZE	3.9	29.2	−15.3	ZZ
**14,423**	4.1	21.8	−19.1	ZE	3.4	27.5	−13.6	ZZ
**18,168**	3.7	17.1	−17.0	ZE	3.3	31.8	−13.8	ZZ
**19,729**	3.4	17.1	−17.0	ZE	3.9	43.7	−18.4	ZZ
$\Delta E_{AV}^{‡}$		17.1				23.9		

^a^ H stands for H_12proS_; ^b^ H stands for H_9proR_.

**Table 2:** Number of the MD frame used as initial coordinates for the QM/MM optimization in hALOX12; optimized O-C_14_ distances (in Å) at the reactants and potential energy barriers for the addition reaction as well as the chirality of the peroxyl product and the geometry of the addition approach; potential energy barriers for the rotation of the peroxyl radical’s carbon chain and for the retro-hydrogen abstraction (reorganization of the peroxyl radical and retro-hydrogen abstraction itself). All energies are in kcal/mol.

Frame	$d_{O-C_{14}}^{react}$	$\Delta E_{O_{2}add}^{‡}$	Chirality of Product	Geometry of Addition	$\Delta E_{Crot}^{‡}$	$\Delta E_{reorg}^{‡}$	$\Delta E_{retro-Habs}^{‡}$
**8721**	3.2	3.0	S	Antarafacial	6.6	10.2	33.3
**10,106**	3.2	4.3	S	Antarafacial	5.8	-	-
**18,168**	3.1	3.1	S	Antarafacial	5.9	13.1	20.6

**Table 3:** Optimized H_12proS_-OH^−^ distances (in Å) at reactants, potential energy barriers, and reaction energies (in kcal/mol) for the H_12proS_ abstraction process. The stereochemistry of the pentadienyl group around C_12_ at the product radical is also given. The exponential average potential energy barrier [46] (in kcal/mol) for the H_12proS_-abstraction is included in the last row.

	H_12proS_			
Frame	${d_{H-{OH}^{-}}^{react}\;}^{a}$	$\Delta E^{‡}$	ΔE	Pentadienyl Stereochemistry
**940**	3.2	20.9	−12.5	ZZ
**1548**	3.0	19.8	−11.8	ZZ
**1709**	2.9	24.5	−12.4	ZZ
**2132**	2.8	22.1	−9.7	ZZ
**2515**	3.2	27.2	−8.9	ZZ
**3020**	2.8	19.8	−10.8	ZZ
**3556**	3.2	24.5	−9.9	ZZ
**4502**	2.7	15.9	−16.1	ZZ
**5758**	2.6	20.4	−12.1	ZZ
**9207**	2.6	22.6	−13.1	ZZ
$\Delta E_{AV}^{‡}$		17.6		

^a^ H stands for H_12proS_.

## Data Availability

The data presented in this study are contained within the article.

## Supplementary Materials

The supporting information can be downloaded at: https://www.mdpi.com/article/10.3390/ijms24076064/s1.
