# Supplementary material for: Hydroperoxidation of Docosahexaenoic Acid by Human ALOX12 and pigALOX15-mini-LOX
Source: Int J Mol Sci. 2023 Mar 23;24(7):6064. doi: 10.3390/ijms24076064 (PMC10094721; doi:10.3390/ijms24076064)
Supplement: Supplementary file 1 [file ijms-24-06064-s001.zip › ijms-2249651-supplementary.pdf]

Supplementary Material

Hydroperoxidation of Docosahexaenoic Acid by Human ALOX12 and pigALOX15-mini-LOX

Miquel Canyelles-Niño[1,2], Àngels González-Lafont[1,3,*] and José M. Lluch[1,3]

[1] Departament de Química, Universitat Autònoma de Barcelona, 08193 Bellaterra, Barcelona, Spain;

[2] Arquebio SL, Carrer de Álava 51, 08005 Barcelona, Spain

[3] Institut de Biotecnologia i Biomedicina (IBB), Universitat Autònoma de Barcelona, 08193 Bellaterra, Barcelona, Spain

* Correspondence: angels.gonzalez@uab.cat

Table S1. Number of the MD frame used as initial coordinates for the QM/MM optimization in hALOX12**,** optimized $H_{12proS/proR}$-OH-, $H_{9pros/proR}$-OH-, and $H_{15pros/proR}$-OH- distances (in Å) at reactants, potential energy barriers (in kcal/mol) for the $H_{12proS/proR}$, $H_{9proS/proR}$, and $H_{15proS/proR}$ abstraction processes. The stereochemistry of the pentadienyl radicals centered at C_{12}, C_9, and C_{15} is also given. In the last row, the exponential average potential energy barriers (in kcal/mol) are included for all the H-abstractions.

	**H_{12proS}**			**H_{12proR}**			**H_{9proS}**			**H_{9proR}**			**H_{15proS}**			**H_{15proR}**		
**Frame**	$d^{react}_{H-OH^-}$	$\Delta E^{\ddagger}$	**Penta-dienyl stereochemistry**	$d^{react}_{H-OH^-}$	$\Delta E^{\ddagger}$	**Penta-dienyl stereochemistry**	$d^{react}_{H-OH^-}$	$\Delta E^{\ddagger}$	**Penta-dienyl stereochemistry**	$d^{react}_{H-OH^-}$	$\Delta E^{\ddagger}$	**Penta-dienyl stereochemistry**	$d^{react}_{H-OH^-}$	$\Delta E^{\ddagger}$	**Penta-dienyl stereochemistry**	$d^{react}_{H-OH^-}$	$\Delta E^{\ddagger}$	**Penta-dienyl stereochemistry**
**322**	3.5	15.9	ZE	3.5	27.3	ZZ	4.7	41.1	ZE	3.5	36.4	ZZ	6.0	34.9	ZZ	4.8	31.2	ZE
**2253**	5.4	20.5	ZE	4.4	29.7	ZZ	3.6	29.4	ZE	3.5	23.4	ZZ	7.5	34.4	ZE	5.8	42.8	ZZ
**4986**	5.4	19.3	ZE	4.9	34.6	ZZ	4.5	53.5	ZE	5.0	35.0	ZZ	7.9	31.7	ZE	6.1	43.1	ZE
**6993**	3.9	18.0	ZE	3.5	29.3	ZZ	4.1	39.1	ZE	3.1	29.7	ZZ	6.0	32.5	ZZ	4.7	43.4	ZZ
**8721**	5.0	21.9	ZE	4.3	28.8	ZZ	3.4	34.6	ZE	3.3	22.7	ZZ	7.4	39.6	ZE	5.6	51.4	ZZ
**10106**	5.5	24.3	ZE	4.3	28.5	ZZ	3.7	37.9	ZE	3.4	25.6	ZZ	6.9	36.6	ZE	5.3	43.2	ZE
**12860**	5.2	22.9	ZE	4.5	37.3	ZZ	4.1	40.3	ZE	3.9	29.2	ZZ	7.3	43.2	ZE	5.7	54.7	ZE
**14423**	4.1	21.8	ZE	3.7	29.4	ZZ	3.9	36.3	ZE	3.4	27.5	ZZ	6.3	51.2	ZZ	5.1	44.3	ZZ
**18168**	3.7	17.1	ZE	3.2	23.7	ZZ	4.5	38.5	ZE	3.3	31.8	ZZ	6.0	25.3	ZZ	4.9	31.8	ZE
**19729**	3.4	17.1	ZE	3.0	24.8	ZZ	5.3	46.7	ZE	3.9	43.7	ZZ	4.6	35.1	ZZ	5.4	21.9	ZZ
$\Delta E^{\ddagger}_{AV}$		17.1			25.0			30.7			23.9			26.7			23.2	

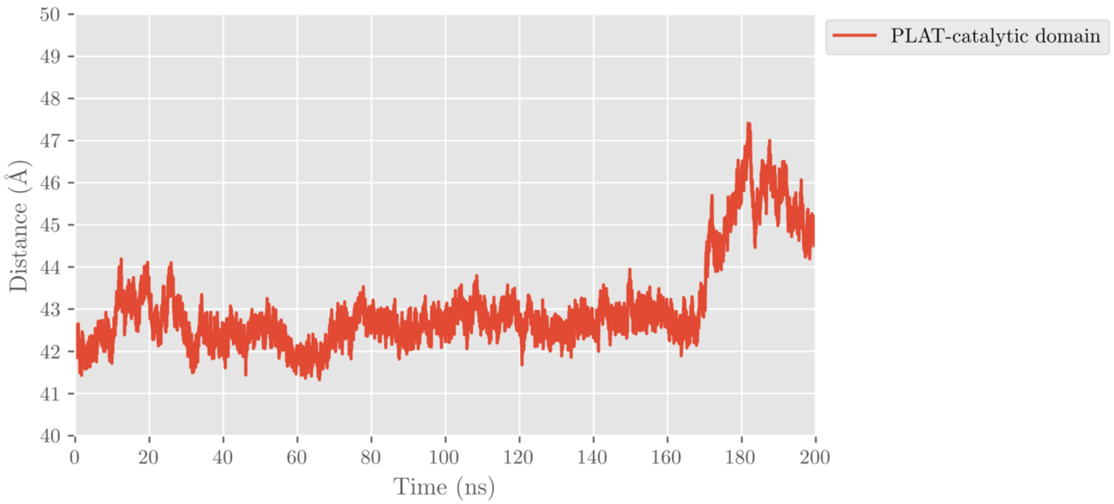

Figure S1. Distance between the geometrical centers of the PLAT and catalytic domains along the MD trajectory for replica 1 of hALOX12.

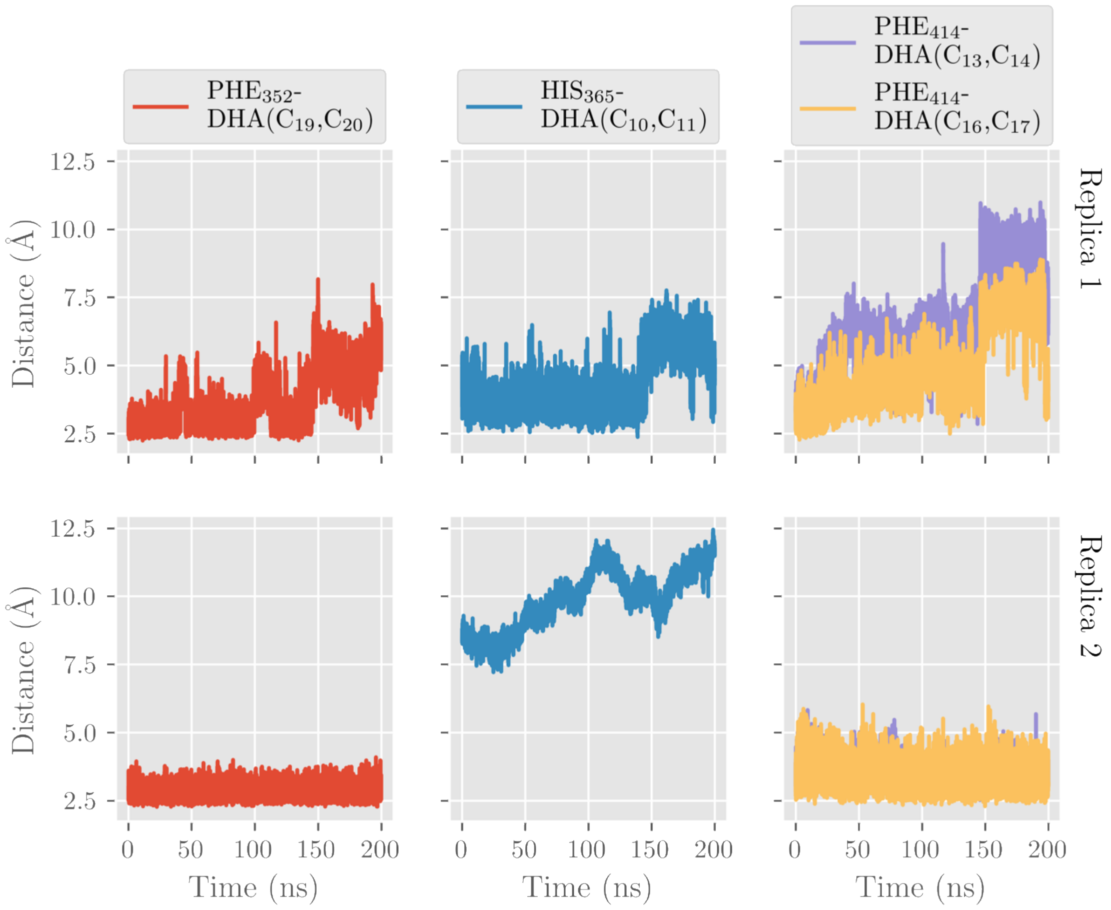

Figure S2. Distances between the side chain of different aromatic residues of hALOX12 and the closest atom of the DHA double bonds versus time for the MD replica 1 and replica 2.

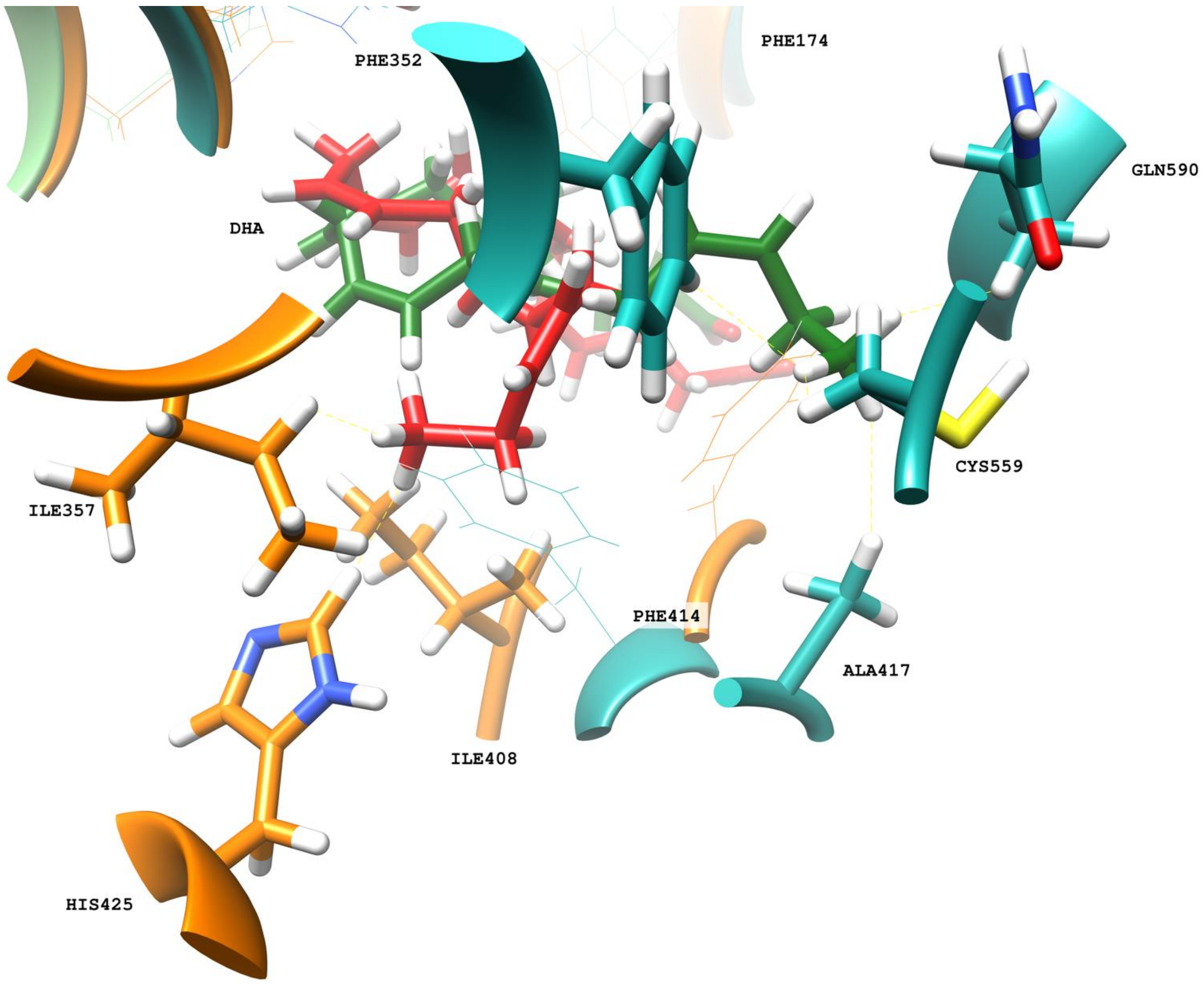

Figure S3. Main interactions between the terminal methyl of DHA and residues at the bottom of hALOX12's cavity for overlayed close and open conformations.

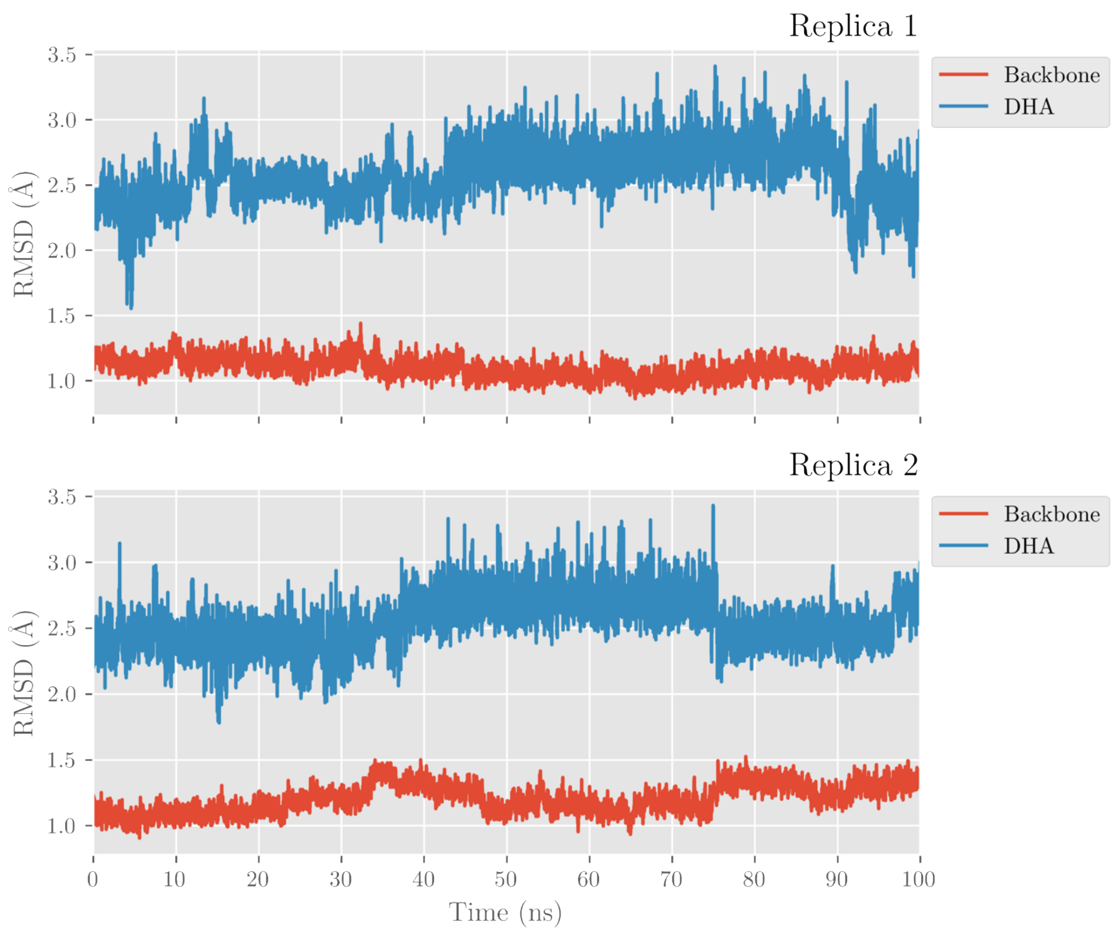

Figure S4. Protein and substrate backbone RMSDs versus time for the MD replica 1 and replica 2 in pigALOX15-mini-LOX.

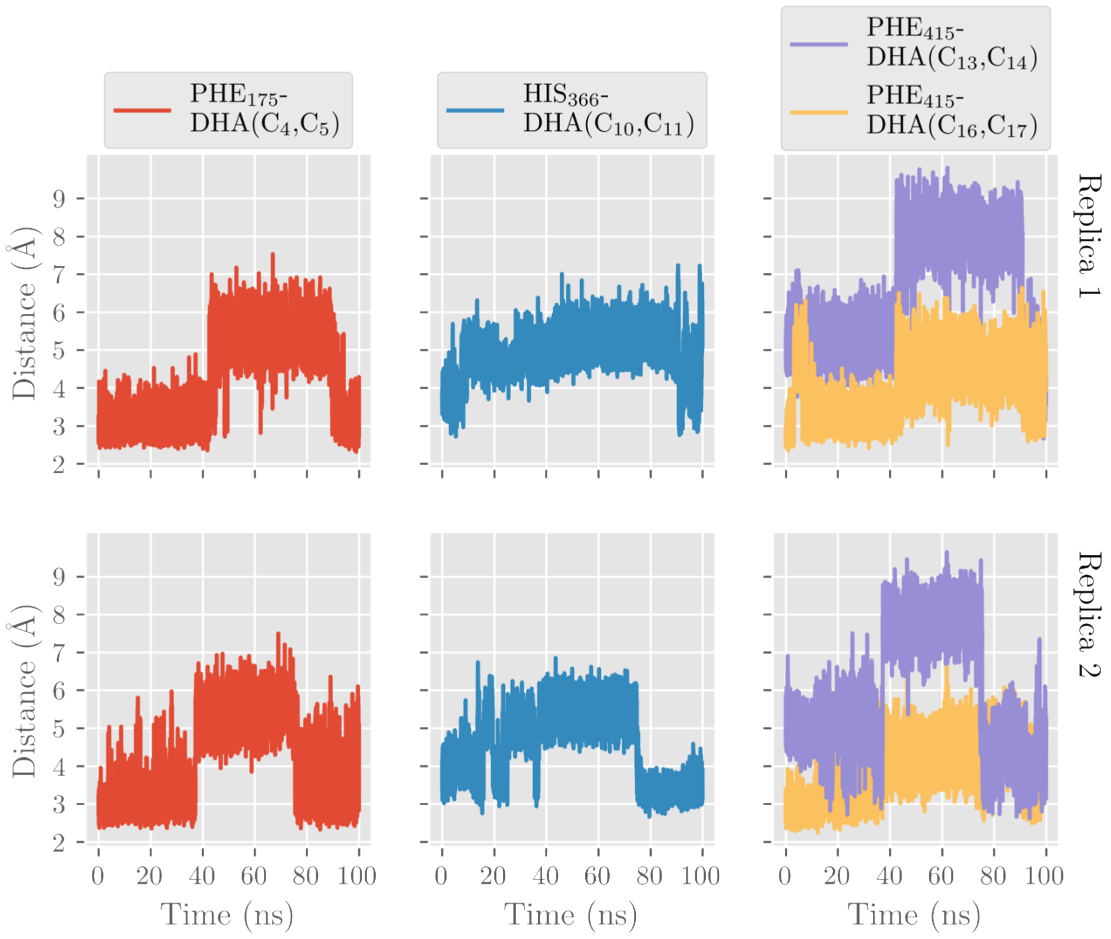

Figure S5. Distances between the side chain of different aromatic residues of pigALOX15-mini-LOX and the closest atom of the DHA double bonds versus time for the MD replica 1 and replica 2.
